# Effects of cellular membranes and the precore protein on hepatitis B virus core particle assembly and DNA replication

**DOI:** 10.1128/mbio.03972-24

**Published:** 2025-03-05

**Authors:** Yu-Chen Chuang, Jing-hsiung James Ou

**Affiliations:** 1 Department of Molecular Microbiology and Immunology, University of Southern California Keck School of Medicine, Los Angeles, California, USA; Johns Hopkins University School of Medicine, Baltimore, Maryland, USA

**Keywords:** hepatitis B virus, core particle assembly, HBV DNA replication, subcellular fractionation, autophagic degradation

## Abstract

Hepatitis B virus (HBV) core particle is critical for the transport and replication of the viral DNA genome. By characterizing HBV core particles in different subcellular compartments, we found that when the HBV core protein was expressed by itself, it formed core particles with a uniform and fast mobility on a non-denaturing agarose gel. However, when the core protein was expressed from a replication-competent 1.3mer HBV genome, it formed particles with more heterogeneous structures. The presence of the precore protein, a protein related to the core protein, led to the formation of chimeric particles in the cytoplasm that consisted of both precore and core proteins. When the precore protein was expressed by itself, it could also form particulate structures in association with cellular RNAs in the nucleus. Our further analysis revealed that, in cells with replicating HBV, only the fast-migrating core particles on the gel contained the viral RNA and DNA, and the membrane-associated core particles contained more mature HBV DNA than the cytosolic core particles. In addition, the precore protein reduced the level of core particle-associated HBV DNA. Interestingly, the precore protein level in the cells could be increased by the degradative autophagy inhibitor bafilomycin A1 but not by the proteasome inhibitor MG132, suggesting that autophagy might regulate the biological activities of the precore protein. In conclusion, our results indicated that membranes and the precore protein could regulate HBV core particle assembly and DNA replication and suggested a role of autophagy in the regulation of HBV precore protein activities.

**IMPORTANCE:**

Hepatitis B virus (HBV) is an important human pathogen that chronically infects 254 million people in the world. This virus contains a core particle, which plays an important role in the transport and replication of the viral DNA genome. The major protein constituent of this particle is the viral core protein. In this report, we examined how the subcellular compartments and the related precore protein might affect the core particle structure and viral DNA replication. We found that the subcellular localizations could affect the core particle assembly, and membranes and the precore protein could regulate HBV DNA replication. We also found that the inhibition of autophagic degradation increased the precore protein level, suggesting a role of autophagy in the regulation of precore protein activities. These findings provided important information for further understanding the HBV life cycle, which will aid in the development of novel drugs for the treatment of HBV patients.

## INTRODUCTION

Hepatitis B virus (HBV) is a hepatotropic virus that can cause severe liver diseases including cirrhosis and hepatocellular carcinoma. As of 2022, there was an estimated 254 million people worldwide who were chronically infected by HBV, resulting in approximately 1 million deaths annually (1). HBV is a small, enveloped DNA virus. Its envelope consists of three co-carboxy-terminal glycoproteins, collectively referred to as the surface antigen (HBsAg). The core of the virus primarily consists of the core protein and the DNA genome. The HBV genome is a partially double-stranded and circular molecule with a length of 3.2 kilobases (kb). It contains four overlapping genes that code for HBsAg, the core protein and a related protein termed the precore protein, the DNA polymerase, and a regulatory protein named the X protein (HBx).

Upon infection of hepatocytes, the HBV core particle delivers the viral genomic DNA to the nucleus. The genomic DNA is subsequently repaired to form the covalently closed circular DNA (cccDNA) (2), which directs the transcription of viral RNAs. One of the viral RNAs is 3.5 kb in length and is larger than the genome size. This RNA codes for the core protein and the viral DNA polymerase. It also serves as the template for synthesizing the HBV genomic DNA and is hence often referred to as the pregenomic RNA (pgRNA). The conversion of the pgRNA into the DNA genome takes place in the core particle and is mediated by the viral DNA polymerase, which is also a reverse transcriptase. This process is initiated by the reverse transcription of the pgRNA into the minus-strand DNA, followed by the synthesis of the plus strand to result in the formation of the relaxed circular DNA (rcDNA) genome (for review of the HBV life cycle, see reference 3).

The core particle may contain 90 or 120 core protein homodimers, yielding particles that exhibit either *T* = 3 or *T* = 4 symmetry, with the latter being the predominant form (4). The core particle displays a conformational epitope, which is serologically known as the HBV core antigen (HBcAg). The coding sequence for core/precore proteins contains two in-frame start codons. The translation initiating from the downstream start codon generates the 21 kDa core protein (p21), and the translation initiating from the upstream start codon generates the 25 kDa precore protein (p25), which is the precursor of the e antigen (HBeAg) found in the sera of HBV patients. The N-terminus of the precore protein encompasses a 19-amino acid signal peptide that forms a complex with the signal recognition particle and guides the nascent precore polypeptide to the endoplasmic reticulum (ER) (5, 6). This signal peptide is removed by the signal peptidase located in the ER lumen, generating the precore protein derivative p22 (6). The C-terminal arginine-rich domain of p22 is subsequently cleaved by a furin-like protease at multiple sites in the *trans*-Golgi network to generate a heterogeneous population of precore protein derivatives (i.e., HBeAg) for secretion (7, 8). A fraction of p22 can also be released back into the cytosol and further transported into the nucleus (9). The cytosolic p22 could serve as a dominant-negative factor in the assembly of the core particle, and its overexpression had been shown to suppress HBV DNA replication both *in vitro* and *in vivo* (10, 11). Chimeric particles consisting of the core protein and the cytosolic p25 and p22 precore proteins had been reported (12, 13). The precore protein is not essential for HBV replication, and a mutation of G to A at nucleotide 1896 located between the two in-frame start codons (i.e., the precore sequence), which generates a premature stop codon that abolishes the expression of the precore protein but not the core protein, was frequently detected in chronic HBV patients (14).

Autophagy is a catabolic process that allows the cells to remove protein aggregates and damaged organelles. HBV infection can activate the early autophagic pathway to support its DNA replication both *in vitro* and *in vivo* (15, 16). The process of autophagy begins with the generation of a membrane crescent known as the phagophore. Phagophores will grow to become an enclosed double-membrane structure known as the autophagosome. HBV core protein can directly interact with autophagy-related protein 12 (ATG12) (17), which is part of the ATG5–ATG12–ATG16L complex necessary for the formation of phagophores. HBV core particles are also peripherally associated with autophagosomes (18). Autophagosomes mature by fusing with lysosomes to form autolysosomes, wherein the cargoes of autophagosomes are digested by lysosomal enzymes in a process known as “degradative autophagy.” Autophagosomes can also fuse with multivesicular bodies (MVBs) to form amphisomes, which can fuse with plasma membranes to release their cargoes through a process called “secretory autophagy.” HBV was thought to also use secretory autophagy to promote its envelopment and eventual release (17–19).

In this report, we studied HBV core particles in different subcellular compartments and how they might be affected by the precore protein. We found that the precore protein affected the structure of the core particles in both the cytoplasm and the nucleus, and membranes also affected the assembly of the core particles. Moreover, both the precore protein and membranes also affected the HBV DNA replication. Interestingly, we also found that the precore protein level could be increased by bafilomycin A1 (BafA1), an inhibitor of degradative autophagy, implicating a role of autophagy in the regulation of HBV precore protein activities.

## RESULTS

### Characterization of HBV core particles in both the nucleus and the cytoplasm

**Fig 1:**
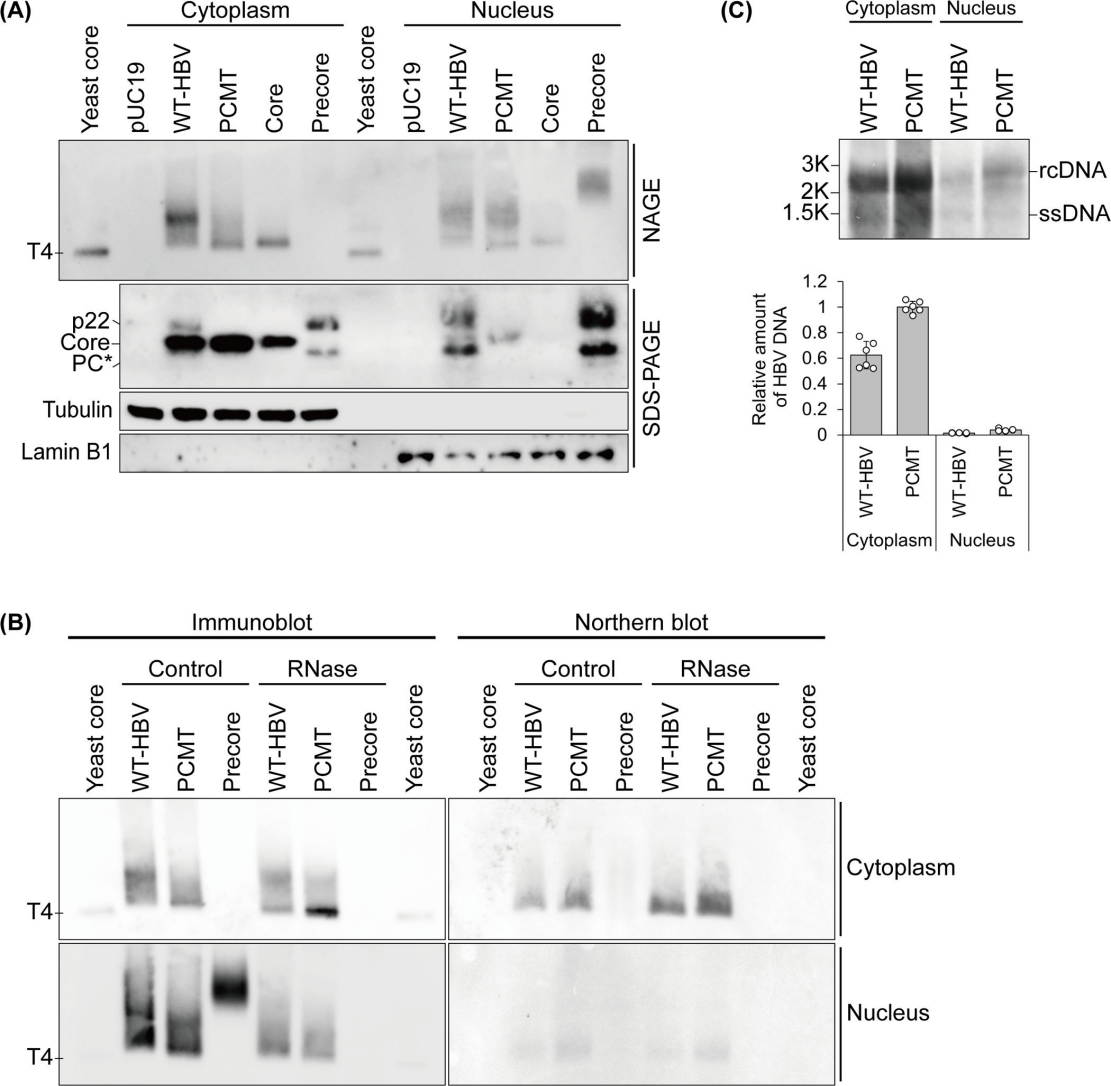
Analysis of HBV core particles in both the cytoplasm and the nucleus. (A) Huh7 cells transfected with the control pUC19 vector, the 1.3mer wild-type HBV genomic DNA (WT-HBV), the precore-negative HBV mutant (PCMT), the core-expressing plasmid, or the precore-expressing plasmid were lysed with a hypotonic buffer, followed by a brief centrifugation to separate the cytoplasmic fraction and the nuclear fraction, which were then treated with 0.6% NP-40 and analyzed for core particles by non-denaturing agarose gel electrophoresis (NAGE) followed by immunoblot analysis using the anti-HBcAg antibody. The recombinant core particle produced in the yeast was used as a marker. Both cytoplasmic and nuclear fractions were also subjected to immunoblot analysis for precore and core proteins, tubulin, and lamin B1 using SDS-PAGE. PC*, a precore protein derivative. (B) The cytoplasmic fraction (top panels) and the nuclear fraction (bottom panels) with and without the RNase treatment were subjected to NAGE followed by immunoblot analysis for core particles (left panels) or Northern blot analysis for particle-associated HBV RNAs. (C) The HBV DNA packaged in the core particles in both the cytoplasmic fraction and the nuclear fraction was analyzed by Southern blot. The relative levels of HBV in the cytoplasmic fraction and the nuclear fraction were quantified by ImageJ and shown in the bar chart, which took into consideration that only 10% of the cytoplasmic fraction and 100% of the nuclear fraction were used for the Southern blot analysis. The PCMT DNA level in the cytoplasm was arbitrarily defined as 1.

To investigate the possible effect of the precore protein on the assembly of core particles, we transfected Huh7 cells, a human hepatoma cell line, with the plasmid that contained the 1.3mer overlength wild-type HBV genome (WT-HBV) or the 1.3mer HBV precore mutant (PCMT) that was incapable of expressing only the precore protein. The vector pUC19 was used as the control. Additionally, the plasmid that expressed only the core protein or the precore protein was used in the transfection studies for comparison. Cells were lysed with a hypotonic buffer 2 days after DNA transfection and separated into the cytoplasmic fraction and the nuclear fraction. The successful separation of these two fractions was confirmed by immunoblot analysis of tubulin and lamin B1, which are markers of the cytoplasm and the nucleus, respectively (Fig. 1A). While the core protein was detected primarily in the cytoplasm, a significant amount of the precore protein derivatives, including p22, was detected in the nuclear fraction (Fig. 1A). The detection of the precore protein derivatives in the nuclear fraction was not surprising, as previous studies indicated that the precore protein derivative p22 could be transported into the nucleus after the removal of its signal peptide (20). After the solubilization of cellular membranes with the nonionic detergent Nonidet P-40 (NP-40), core particles in both the cytoplasmic fraction and the nuclear fraction were analyzed on a non-denaturing agarose gel (i.e., the “particle gel”) and detected by an antibody directed against HBcAg. The recombinant core particle that was produced in the yeast was used as the marker (see Fig. S1). As shown in Fig. 1A, the core particles generated by the core protein alone in both the cytoplasm and the nucleus migrated as one band with an electrophoretic mobility slightly less than that of the yeast core particles. The reason why the core particles produced in the yeast migrated faster than those produced in Huh7 cells is unclear and might be caused by host factors. A fast-migrating core particle band could also be detected in both the cytoplasmic fraction and the nuclear fraction of WT-HBV- and PCMT-transfected cells. This core particle band of WT-HBV migrated slightly more slowly than that of PCMT, suggesting an effect of the precore protein on the structure of the core particle. A strong core particle signal with a reduced mobility on the gel was also detected in the cytoplasmic fraction of WT-HBV-transfected cells but not in that of PCMT-transfected cells. This core signal likely represented the chimeric core particles that consisted of both the precore protein and the core protein, as previously reported (12, 13) (also see below). The signal of these putative chimeric particles was partially diminished if the cytoplasmic fraction was treated with RNase and, interestingly, this signal was not detected by the HBV antisense riboprobe in the Northern blot analysis (Fig. 1B). These results indicated that a significant fraction of these particles were empty particles, and the rest of them were loosely held together by cellular RNAs and hence were lost once the RNAs were removed by RNase. Similarly, in the nuclear fraction of both WT-HBV- and PCMT-transfected cells, in addition to the fast-migrating core particle band, a diffuse core signal with reduced electrophoretic mobility could also be detected (Fig. 1A). This signal was also diminished by the RNase treatment and could not be detected by the HBV antisense riboprobe in the Northern blot analysis (Fig. 1B). In contrast, the signal of the fast-migrating core particles in the cytoplasm of WT-HBV- and PCMT-transfected cells was not affected by RNase, and those in the nucleus were partially reduced by the RNase treatment (Fig. 1B). These fast-migrating core particles could be detected by the antisense HBV riboprobe in the Northern blot analysis (Fig. 1B), indicating that they contained the HBV pgRNA.

To determine whether the core particles that we detected in the cytoplasm and the nucleus of Huh7 cells could also be detected in other cell lines, we analyzed the HBV core particles in HepAD38 cells. HepAD38 is a stable cell line that was derived from HepG2 cells, a human hepatoblastoma cell line. HepAD38 contains an integrated HBV genomic DNA under the expression control of the cytomegalovirus (CMV) promoter and tetracycline (21). As shown in Fig. S2A, a fast-migrating core particle band could be detected in both the cytoplasmic fraction and the nuclear fraction. In addition, a diffuse and slower-migrating core particle band was also detected in the nuclear fraction. This result is similar to what we observed in Huh7 cells that were transfected with the PCMT DNA. Indeed, unlike Huh7 cells transfected with the WT-HBV DNA, our immunoblot analysis revealed little precore protein signals in both the cytoplasmic fraction and the nuclear fraction (Fig. S2A). This result was consistent with a previous report, which indicated that HepAD38 cells could only produce HBeAg after a prolonged incubation and the accumulation of cccDNA (22). The lack of expression of HBeAg was further confirmed when we harvested the incubation media of HepAD38 cells and conducted the enzyme-linked immunosorbent assay (ELISA) analysis. As shown in Fig. S2B, in contrast to Huh7 cells transfected with the WT-HBV DNA, the incubation media of HepAD38 cells contained little HBeAg in our experimental conditions. As the HBV DNA used in our transfection studies belonged to genotype A and that in HepAD38 cells belonged to genotype D, these results indicated that the assembly of HBV core particles was not affected by cell types and HBV genotypes.

In contrast to the cells transfected by the core protein-expressing plasmid, cells transfected by the precore protein-expressing plasmid generated little core particle-like structures in the cytoplasm (also see below), although a diffuse signal with much reduced electrophoretic mobility was detected by the anti-HBcAg antibody in the nuclear fraction (Fig. 1A). This signal was lost upon the RNase treatment and could not be detected by the HBV antisense riboprobe (Fig. 1B). As the precore-expressing plasmid did not produce the core protein (see the immunoblot result in Fig. 1A) nor the pgRNA, this structure likely represented a precore protein structure that was held together by cellular RNAs to display the HBcAg antigenic determinant. Hereafter, it will be referred to as “precore particles.”

Next, we performed Southern blot analysis to characterize the HBV DNA that might be associated with core particles. As shown in Fig. 1C, there was no qualitative difference between the HBV DNA in the cytoplasmic fraction and the nuclear fraction, although quantitatively, the latter contained a much lower level of the HBV DNA (i.e., ~2%–4% of that in the cytoplasm). In addition, the PCMT generated a slightly higher level of HBV DNA than the WT-HBV DNA. This result was consistent with the previous reports (12, 13, 23), which indicated that the precore protein and its derivatives could serve as a dominant-negative factor to suppress HBV nucleocapsid assembly and DNA replication.

In summary, our results shown in Fig. 1 indicated that, first, core particle structures could be detected in both the cytoplasm and the nucleus; second, the precore protein affected the assembly of the core particles, leading to the production of empty particles and particles that were associated with cellular RNAs; third, the precore protein in association with cellular RNAs could form precore particles in the nucleus; fourth, the fast-migrating core particles on the gel were nucleocapsids that contained HBV pgRNA; and fifth, a small but detectable amount of HBV pgRNA and DNA were associated with the core particles in the nucleus.

### Characterization of HBV core particles associated with membranes and in the cytosol

**Fig 2:**
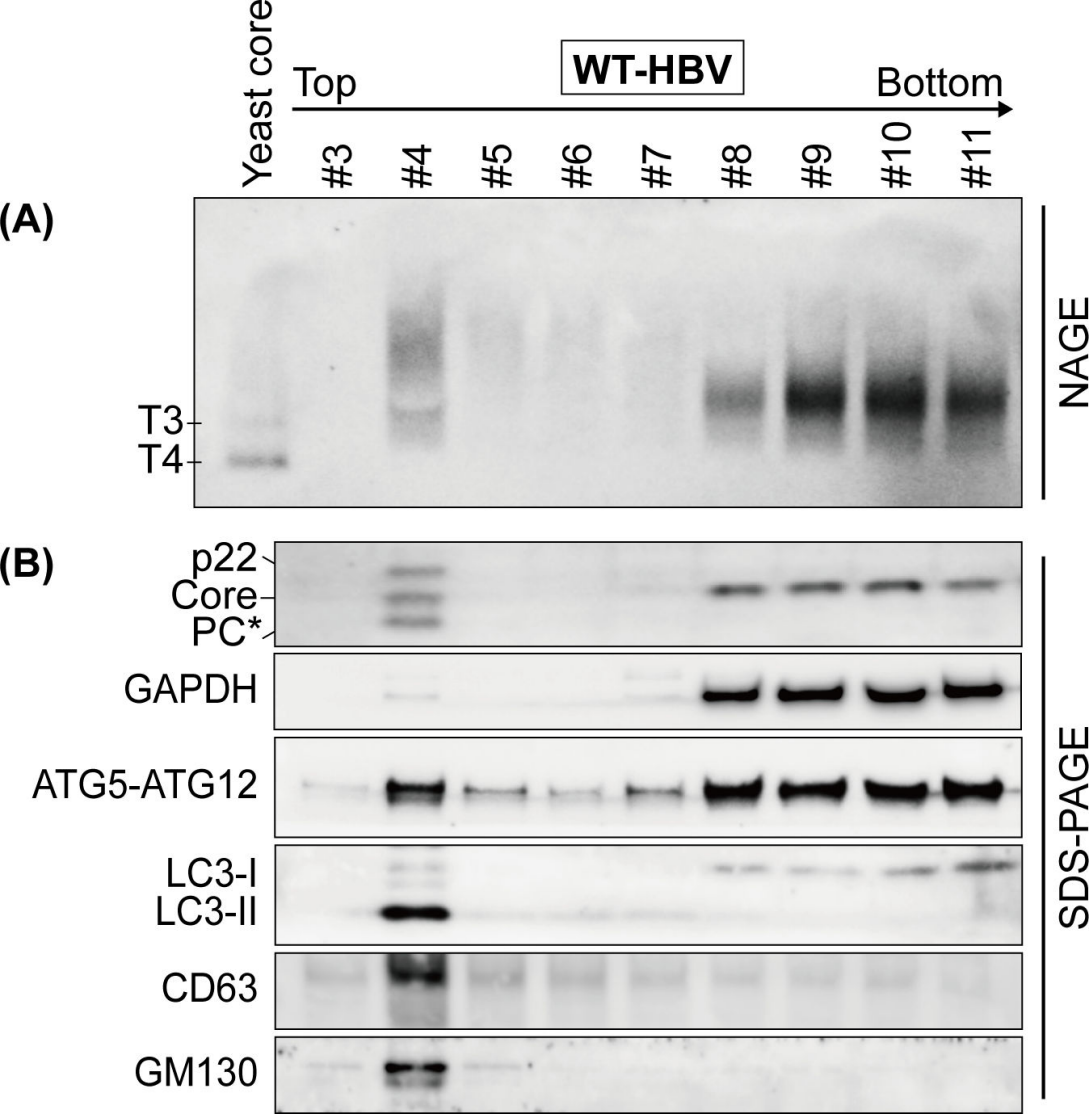
Analysis of the HBV particles in membrane and cytosolic fractions. The cytoplasmic fraction of WT-HBV DNA-transfected cells was further fractionated using a discontinuous sucrose gradient. Individual sucrose gradient fractions were then analyzed by non-denaturing agarose gel electrophoresis (NAGE) (A) or subjected to immunoblot analysis for the proteins indicated (B). The recombinant yeast core particles were used as the maker. Note that a small amount of yeast core particles that might represent the *T* = 3 particles could sometimes be detected.

Previous studies indicated that autophagic membranes could serve as a platform for the assembly of HBV core particles (17–19). To further determine the relationship between membranes and core particles, we conducted the membrane flotation assay using a discontinuous sucrose gradient to separate the cytoplasm into membrane and cytosolic fractions (Fig. 2A). The cytoplasmic lysates of Huh7 cells transfected with WT-HBV were used for this fractionation study. As shown in Fig. 2B, lipidated LC3 (i.e., LC3-II), CD63, and GM130, which are markers of autophagosomes, MVBs, and the Golgi complex, respectively, were detected almost exclusively in fraction 4 of the sucrose gradient. In contrast, the nonlipidated LC3 (i.e., LC3-I) and glyceraldehyde-3-phosphate dehydrogenase (GAPDH), which are both cytosolic proteins, were detected almost entirely in fractions 8–11. The ATG5-ATG12 heterodimer, which is associated with phagophores and also present in the cytosol (18), was detected in fraction 4 and fractions 8–11. These results indicated that fraction 4 was the membrane fraction and fractions 8–11 were the cytosolic fractions. In this fractionation study, precore protein derivatives were found only in the membrane fraction, whereas the core protein was detected in both the membrane and the cytosolic fractions. We then analyzed the core particles in individual fractions using the particle gel. As shown in Fig. 2A, the core particles in the cytosolic fractions were prominent and uniform in mobility. This core particle band was apparently the major core particle band detected in the cytoplasmic fraction shown in Fig. 1A and consisted of empty core particles and core particles that were apparently associated with cellular RNAs (Fig. 1B). In contrast, the core particle signals detected in the membrane fraction were weak and heterogeneous in mobility. These membrane-associated core particles could be separated into two groups, with the first group migrating faster than the cytosolic core particles and the second group migrating more slowly. These results indicated that core particles associated with membranes likely had structures distinct from those in the cytosol.

### Alteration of core particle structures by the precore protein

**Fig 3:**
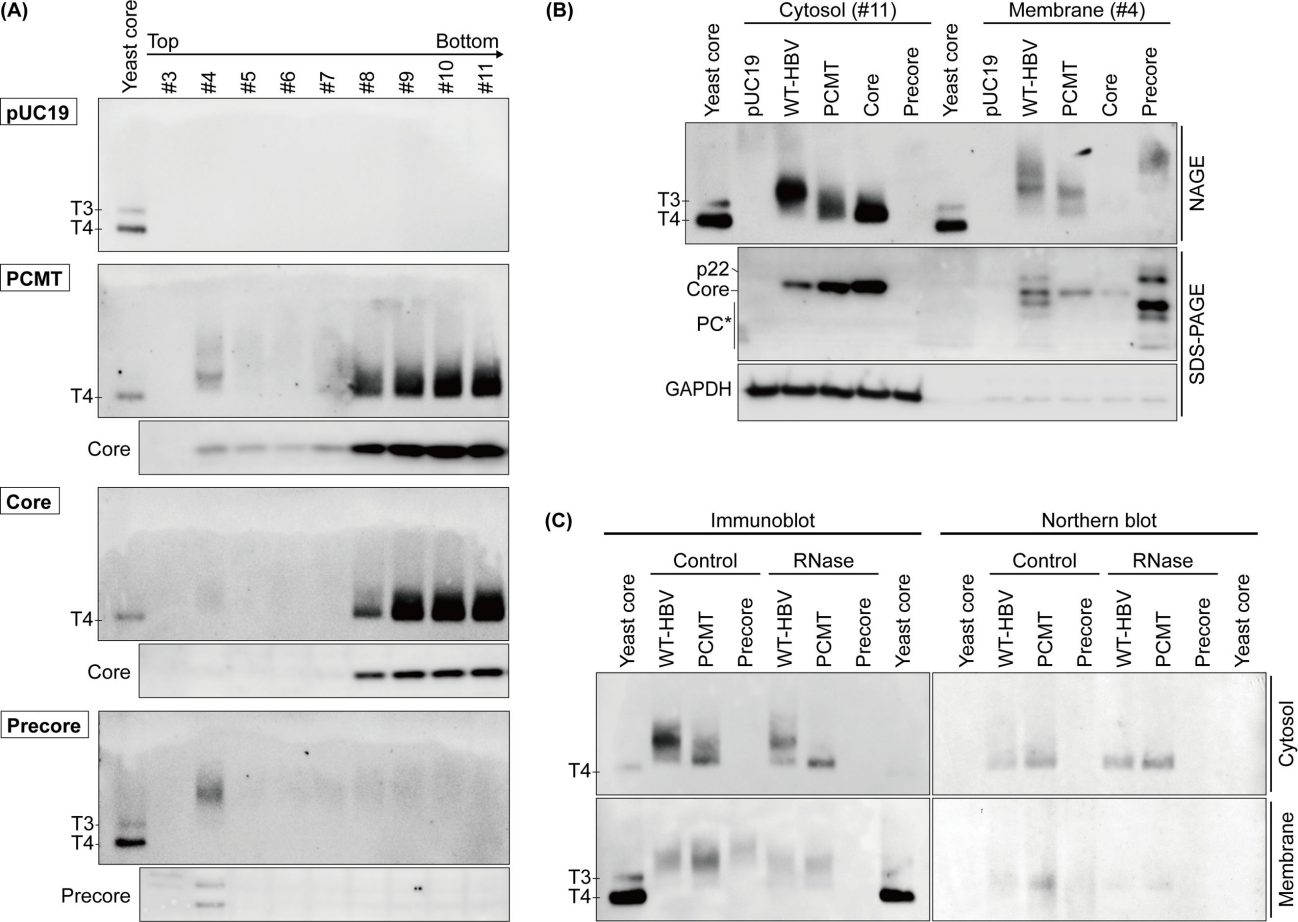
Comparison of the core particles generated by various HBV DNA constructs in the membrane fraction and the cytosolic fraction. (A) The cytoplasmic fraction of Huh7 cells transfected by the control vector pUC19, PCMT, the core-expressing plasmid, or the precore-expressing plasmid was further fractionated by the sucrose gradient as shown in Fig. 2A and analyzed by the particle gel and immunoblot. (B) Comparison of the core particles produced by various HBV DNA constructs. Fraction 4 (i.e., the membrane fraction) and fraction 11 (i.e., the cytosolic fraction) were used for the comparison. (C) Both membrane (bottom panel) and cytosolic (top panel) fractions with and without the RNase treatment were subjected to non-denaturing agarose gel electrophoresis (NAGE) and immunoblot for core particle analysis.

To determine the possible effect of the precore protein on the assembly of the core particles associated with membranes and those in the cytosol, the cytoplasmic fraction of Huh7 cells transfected with the control vector pUC19, PCMT DNA, the core protein-expressing plasmid, or the precore protein-expressing plasmid was also fractionated for the separation of membranes and the cytosol. The immunoblot analysis of the core particles, the precore protein, and the core protein in individual sucrose fractions is shown in Fig. 3A. Similar to WT-HBV-transfected cells, the cytosolic core particles of Huh7 cells transfected by the PCMT DNA migrated as one major band on the gel, whereas those associated with membranes were minor and had more heterogeneous mobilities. The same result was obtained with HepAD38 cells (Fig. S2C). For cells transfected by the core-expressing plasmid, the core particles in the cytosolic fractions also migrated uniformly on the gel, but the core particle signal associated with membranes was barely detectable. The precore protein by itself did not generate obvious signals in the cytosolic fractions. However, a signal with significantly reduced mobility was detected in the membrane fraction (Fig. 3A). This result indicated that the precore protein by itself could also form particulate structures that displayed the HBcAg antigenic determinant when it was associated with membranes. To further compare the structures of the core particles generated by different DNA constructs, we focused our attention on fraction 11 (i.e., the cytosolic fraction) and fraction 4 (i.e., the membrane fraction). As shown in Fig. 3B, top panel, when the cytosolic core particles were compared, the core particles of WT-HBV migrated more slowly on the gel than the core particles of PCMT, which also migrated slightly more slowly than the core particles generated by the core protein alone. These results indicated that the presence of the precore protein could affect the core particle structure, presumably forming a chimeric particle with the core protein as mentioned above. No precore particles could be detected in the cytosolic fraction when the precore protein was expressed by itself. When the membrane-associated core particles were compared, both WT-HBV and PCMT samples generated a heterogeneous population of signals, likely representing assembly intermediates. In addition, diffuse core particle signals with significantly reduced mobilities were detected with the WT-HBV sample, but not with the PCMT sample. These signals migrated between the chimeric core particles and the precore particles and likely represented chimeric particles with increased proportions of the precore protein. When the core protein was expressed by itself, no core particles were detected in the membrane fraction. However, this was likely due to the sensitivity of this particular study, as a weak core particle signal was detected in the membrane fraction in Fig. 3A and core particles had previously been found to be associated with autophagic membranes (18, 24). In addition, a small amount of the core protein could be detected in the immunoblot analysis (Fig. 3B, middle panel). When the precore protein was expressed by itself, no precore particles could be detected in the cytosol, although they could be detected in the membrane fraction (Fig. 3B, top panel).

We also conducted the immunoblot analysis on precore and core proteins. As shown in Fig. 3B, middle panel, the core protein was more efficiently associated with membranes when it was expressed from the 1.3mer genome than when it was expressed by itself. This result indicated a role of the HBV genome in promoting the association of the core protein with membranes. In addition, the association of the core protein with membranes was also slightly enhanced in the presence of the precore protein (i.e., WT-HBV vs PCMT), in spite of the observation that there was a much lower level of the core protein in the cytosolic fraction of cells transfected with the WT-HBV DNA. This result indicated that the precore protein could enhance the association of the core protein with membranes, likely due to its participation in the formation of chimeric particles.

We had also treated the core particles in both the cytosolic fraction and the membrane fraction with RNase. As shown in Fig. 3C, the fast-migrating core particles in both the cytosolic and the membrane fractions were resistant to RNase and positive for HBV RNA in the Northern blot analysis, indicating that they were nucleocapsids that contained the pgRNA. The pgRNA signal of membrane-associated core particles was reduced by RNase (Fig. 3C, bottom right panel), indicating that some of the pgRNA in these membrane-associated core particles was not fully packaged. In contrast to the fast-migrating core particles, the slower-migrating core particles in both the cytosolic fraction and the membrane fraction were partially sensitive to RNase and negative for HBV RNA, indicating that they consisted of empty particles and particles that were associated with and stabilized by cellular RNAs.

### Effect of precore protein on core particle assembly when it was provided in *trans*

**Fig 4:**
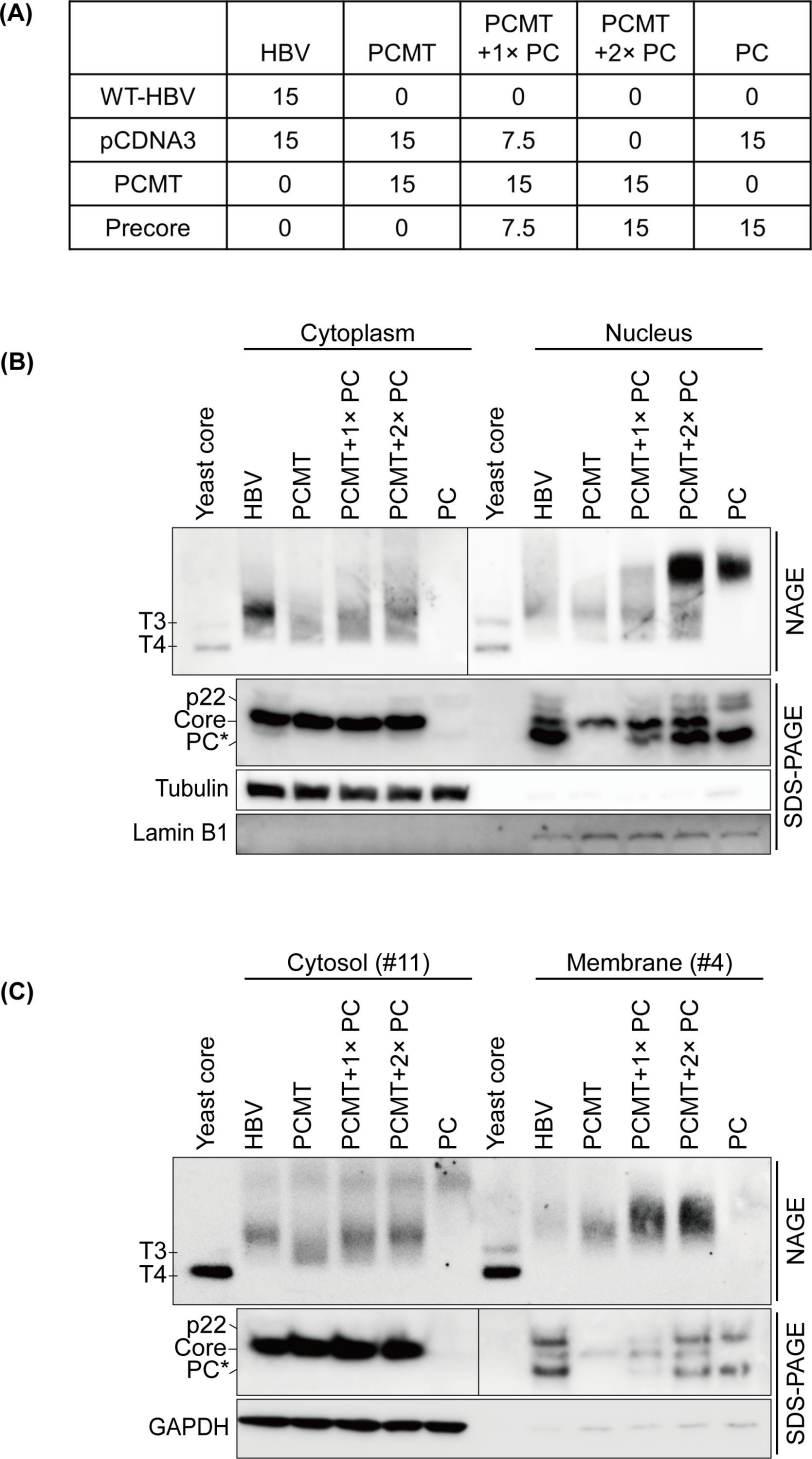
*trans*-complementation analysis for the effect of the precore protein on core particles. (A) Illustration of the *trans*-complementation experimental design. Huh7 cells in 10 cm dishes were co-transfected with the DNA plasmids indicated. PC, the precore-expressing plasmid. Numbers indicated μg DNA used in the co-transfection experiments. (B) Huh7 cells transfected by different combinations of DNA constructs were separated into the cytoplasmic fraction and the nuclear fraction for particle gel and immunoblot analyses. (C) The cytoplasmic fraction was further fractionated into the cytosolic fraction and the membrane fraction for analysis as shown in Fig. 3B.

To further analyze the effect of the precore protein on the core particles, we conducted a *trans*-complementation assay by co-transfecting Huh7 cells with PCMT and an increasing amount of the precore protein-expressing plasmid (Fig. 4A). The core particles and the expression of precore and core proteins were then analyzed by subcellular fractionation experiments. Cells transfected with the WT-HBV DNA were used as the control. As shown in Fig. 4B, in the cytoplasmic fraction, the increase of the precore protein-expressing plasmid in the *trans*-complementation experiment led to an increased appearance of a particle structure that co-migrated with the major core particle band observed in WT-HBV-transfected cells. When the cytoplasmic fraction was further separated into the cytosolic fraction and the membrane fraction, a similar upshift of the core particle band by the precore protein was also observed in both the cytosolic fraction and the membrane fraction (Fig. 4C). These results provided a compelling argument that the precore protein and the core protein could form chimeric particles both in the cytosol and in association with membranes and that the major core particle band that we observed in the cytoplasmic fraction of WT-HBV-transfected cells indeed represented chimeric particles. Interestingly, in the nuclear fraction, the co-transfection of the precore plasmid with PCMT did not alter the mobility of the core particles on the gel (Fig. 4B). Rather, it led to the appearance of a band that co-migrated with precore particles generated by the precore protein alone. This result raised the possibility that the precore protein derivatives that were transported into the nucleus formed the precore particles without interacting with the core protein. Note that the precore particles were inapparent in the nuclear fraction of WT-HBV-transfected cells even though a comparable level of precore protein derivatives was detected in the nuclear fraction (Fig. 4B, lane HBV vs lane PCMT + 2 × PC). This result indicated that although the precore protein provided in *trans* could affect the assembly of the core particles in the cytoplasm, it preferentially formed precore particles without the interaction with the core protein in the nucleus. Note that although the precore protein bands in the cytoplasmic fraction were inapparent in Fig. 4B, they could be detected in the immunoblot with a longer exposure (Fig. S3).

### Enhancement of HBV plus-strand DNA synthesis by membranes

**Fig 5:**
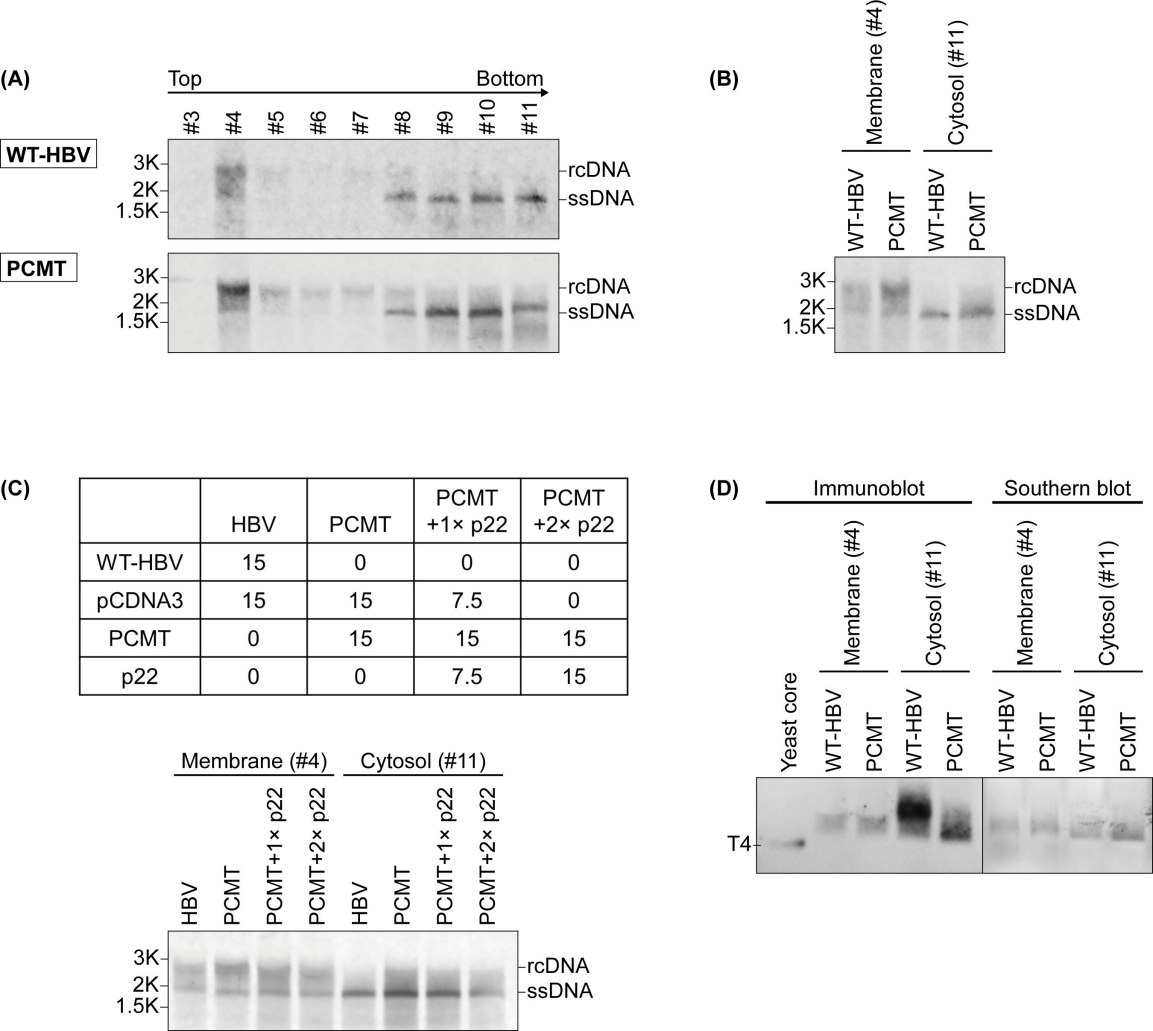
Analysis of core particle-associated HBV DNA in the cytosol and membranes. (A) Southern blot analysis of the core particle-associated HBV DNA. The cytoplasmic fraction of Huh7 cells that were transfected with WT-HBV DNA or the PCMT DNA was further fractionated on a sucrose gradient and analyzed for HBV DNA by Southern blot. (B) Southern blot analysis of the core particle-associated HBV DNA in the membrane fraction (fraction 4) and the cytosolic fraction (fraction 11) of cells transfected with either WT-HBV or PCMT DNA. (C) Southern blot analysis of the core particle-associated HBV DNA in the membrane fraction and the cytosolic fraction of cells co-transfected with PCMT and different amounts of p22-expressing plasmid. Numbers in the table indicated μg DNA used for co-transfection. Cells transfected with WT-HBV DNA were used as the control for comparison. (D) Left panel, immunoblot analysis of core particles in the membrane fraction and the cytosolic fraction using the anti-HBcAg antibody; right panel, Southern blot analysis of HBV DNA in core particles.

Since HBV DNA replication takes place in the core particle, we further characterized HBV DNA in the core particles in individual sucrose gradient fractions by conducting the Southern blot analysis. As shown in Fig. 5A, core particles in the cytosolic fractions (i.e., fractions 8–11) contained primarilysingle-stranded DNA (ssDNA), whereas those in the membrane fraction contained the more mature rcDNA. Similar results were obtained with either WT-HBV or PCMT. The analysis of HepAD38 cells also revealed a similar result, with the membrane fraction containing the more mature HBV DNA than the cytosolic fractions (Fig. S2D). These results indicated that membranes could promote the plus-strand DNA synthesis of HBV and were consistent with our previous finding (18). A direct comparison of the core particle-associated HBV DNA isolated from cells transfected with either WT-HBV or PCMT DNA revealed a slightly higher level of HBV DNA in both the cytosolic fraction and the membrane fraction of PCMT-transfected cells than in WT-HBV DNA-transfected cells, indicating a negative effect of the precore protein on HBV DNA replication (Fig. 5B). To further determine the effect of the precore protein on HBV DNA replication, we conducted the *trans*-complementation experiment by co-expressing p22 (i.e., the precore protein without its signal peptide) with PCMT in Huh7 cells. As shown in Fig. 5C, p22 reduced the ssDNA level in the cytosol in a dose-dependent manner. Although its effect on the ssDNA in the membrane fraction was inapparent, it reduced the level of the rcDNA. These results indicated that p22 could suppress the synthesis of the first-strand HBV DNA in the cytosol and the synthesis of the second-strand DNA when the core particles were associated with the membranes.

As there were multiple forms of core particles in both the cytosolic fractions and the membrane fraction of WT-HBV- and PCMT-transfected cells, we conducted Southern blot analysis on the core particles that were separated by the particle gel. Interestingly, as shown in Fig. 5D, comparing the immunoblot to the left with the Southern blot to the right, HBV DNA was associated with only the fast-migrating core particles in the gel. This was consistent with our results shown in Fig. 1B, which indicated that only the fast-migrating core particles contained the HBV pgRNA. In addition, in Fig. 5D, the DNA signals of membrane-associated core particles migrated slightly more slowly in the gel than their cytosolic counterparts. This might be due to the difference in their DNA contents, as the membrane-associated core particles contained the rcDNA whereas the cytosolic core particles contained primarily the ssDNA.

### Increase of precore protein by inhibition of degradative autophagy

**Fig 6:**
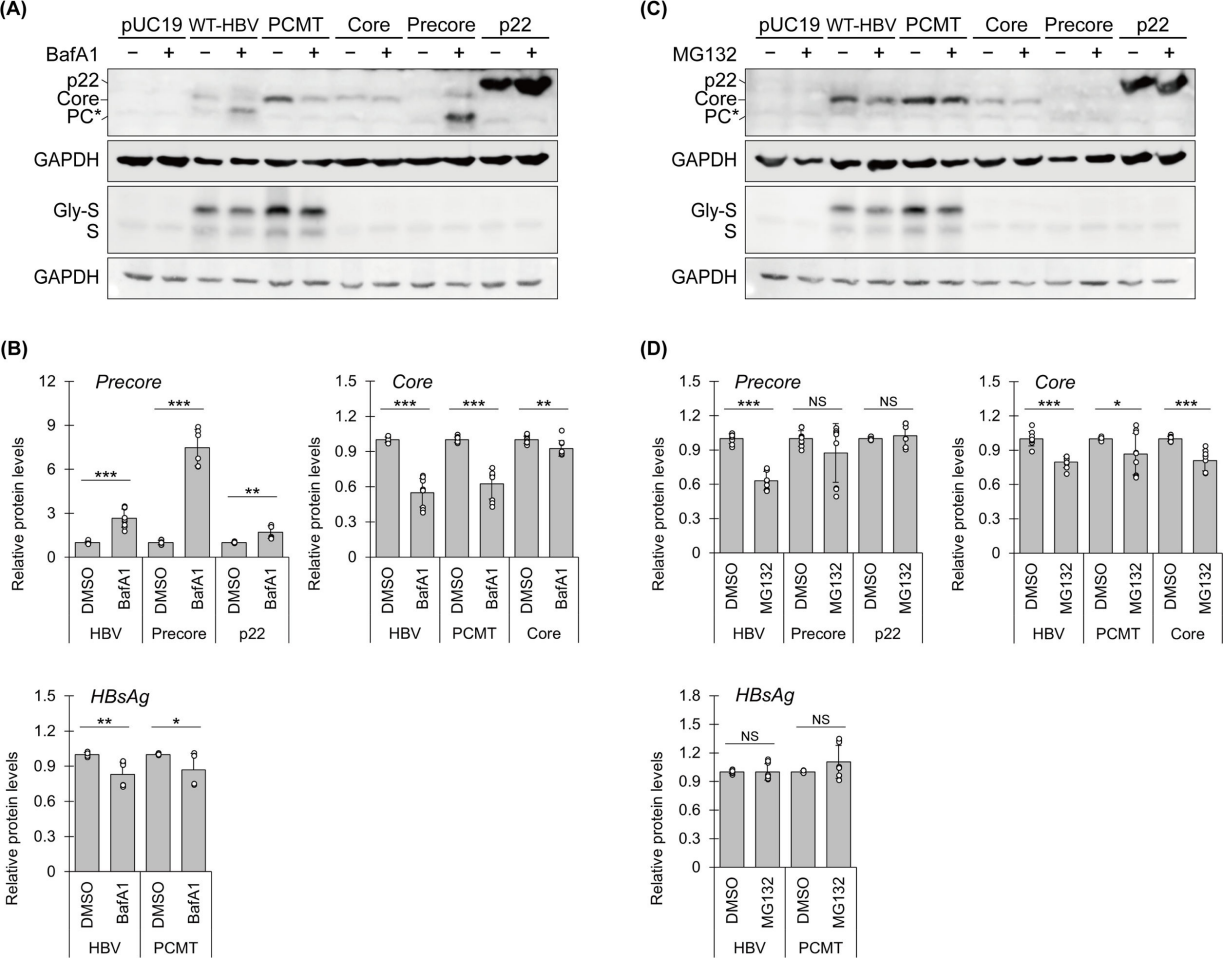
BafA1 but not MG132 increased the precore protein levels in cells. Huh7 cells transfected with various DNA constructs were treated with dimethyl sulfoxide (DMSO), BafA1 (A and B), or MG132 (C and D) as described in Materials and Methods. Cells were then lysed for immunoblot analysis. Note that p22 expressed without its signal peptide migrated slightly more slowly than the p22 derived from the precore construct due to the presence of an additional methionine residue at its N-terminus. (B and D) The relative levels of core and precore protein derivatives and HBsAg with and without drug treatments were quantified by ImageJ and normalized against the GAPDH loading control. The protein levels of DMSO-treated cells were arbitrarily defined as 1. Gly-S and S represent glycosylated and non-glycosylated small surface proteins, respectively.

We previously reported that precore protein derivatives were peripherally associated with autophagosomes (18). That previous finding prompted us to examine the possible effect of autophagy on precore and core proteins. Huh7 cells transfected with WT-HBV, PCMT, or the expression plasmid of the core protein, the precore protein, or the precore protein without its signal peptide (i.e., p22) were treated with BafA1, a vacuolar ATPase inhibitor that suppresses the autophagic protein degradation (25). As shown in Fig. 6A, the levels of precore protein derivatives expressed by WT-HBV and the precore-expressing plasmid as well as p22 expressed by the p22-expressing plasmid were all increased by BafA1, suggesting a role of autophagy in the degradation of precore protein derivatives. In contrast, BafA1 reduced the core protein levels expressed by WT-HBV and PCMT and slightly reduced those expressed by the core protein-expressing plasmid. The quantitative analyses of the effect of BafA1 on precore and core proteins are shown in Fig. 6B. BafA1 also reduced slightly, albeit statistically significant, the level of HBsAg expressed by WT-HBV and PCMT (Fig. 6A and B). In contrast, MG132, a proteasome inhibitor (26, 27), did not increase the levels of the precore protein and instead reduced it when it was expressed from the WT-HBV construct (Fig. 6C and D). MG132 slightly reduced the core protein level regardless of the DNA constructs from which it was expressed (Fig. 6C and D). MG132 had no effect on the HBsAg levels.

## DISCUSSION

The HBV core protein packages the viral pgRNA to form the core particle, in which the pgRNA is converted to the viral genomic DNA (28). In this report, we examined HBV core particles in different subcellular compartments and analyzed how the precore protein, which is structurally related to the core protein in primary sequence, might affect the assembly of core particles. We found that HBV core particles could be detected in both the nucleus and the cytoplasm, and the presence of the precore protein could reduce the mobility of the core particles in the particle gel (Fig. 1A), indicative of an effect of the precore protein on the core particle structures. We also found that when the core protein was expressed by itself, only a fast-migrating core particle species could be detected in both the nucleus and the cytoplasm. However, when the core protein was expressed from either the WT-HBV DNA or the PCMT mutant, additional core particle structures with reduced mobility on the gel could be detected in the nucleus (Fig. 1A). These additional core particle structures did not contain HBV RNA (Fig. 1B). However, they were sensitive to RNase digestion, indicating that they were associated with cellular RNAs. This finding is consistent with a previous report, which suggested that HBV core particles could also be associated with cellular RNAs (29). Interestingly, the precore protein also formed particulate structures in the nucleus that were associated with cellular RNAs (Fig. 1B). This finding is consistent with a previous study, which conducted confocal microscopy using the same anti-HBcAg that we used in our study and found that the precore particles could be detected inside the nucleus (30). The biological significance of the finding that both precore and core proteins could bind to cellular RNAs is unclear, and the possibility that this binding may suppress host gene expression cannot be ruled out.

The fast-migrating core particle species detected in both the nucleus and the cytoplasm of WT-HBV and PCMT-transfected cells contained the HBV RNA and were largely resistant to RNase digestion (Fig. 1B), indicating that they were intact capsid particles that contained the HBV pgRNA. The detection of pgRNA-containing core particles in the nucleus is consistent with a recent report, which indicated that the packaging of pgRNA could take place in the nucleus (31). Our further separation of the cytoplasm into membrane and cytosolic fractions also revealed that the fast-migrating core particle species contained the HBV RNA (Fig. 3B). Interestingly, a significant fraction of the HBV RNA associated with the core particles in the membrane fraction was sensitive to RNase digestion (Fig. 3B), indicating that it was associated with immature core particles and hence was not fully protected from RNase digestion. We also analyzed the relative levels of core particle-associated HBV DNA in both the cytoplasm and the nucleus. Our results indicated that a small but detectable amount of core particle-associated DNA was present in the nucleus (Fig. 1C). These DNA-containing core particles in the nucleus might be derived from the pgRNA-containing particles that were assembled in the nucleus, although it is equally possible that they were imported from the cytoplasm. Further studies will be required to distinguish between these two different possibilities.

In addition to the fast-migrating core particles, the expression of the WT-HBV DNA generated a prominent core particle structure in the cytoplasm (Fig. 1A). As this additional core particle structure was not generated by the precore-negative PCMT genome or by the precore protein when it was expressed by itself, it likely represented a chimeric core particle that contained both the core protein and the precore protein as previously suggested (12, 13). This possibility was supported by our observation that it could be generated if the precore protein was provided in *trans* to the precore-negative PCMT mutant (Fig. 4). Chimeric particles were partially resistant to RNase digestion and did not contain HBV RNA (Fig. 1B and 3C), indicating that they consisted of empty particles and particles that were associated with and stabilized by cellular RNAs.

Our detection of a large amount of the precore protein derivative in the nucleus with a size smaller than that of the core protein was surprising, as the nuclear localization signals of p22 are located at its C-terminal arginine-rich domain (32, 33), which were likely lost in this smaller precore protein derivative. The presence of this protein in the nucleus might be due to its small size, which allowed it to be passively transported into the nucleus, or the proteolytic cleavage of p22 in the nucleus. Alternatively, as HBeAg can form dimers (34), it might be piggybacked by p22 as a heterodimer into the nucleus. The reason why different amounts of core protein and precore protein derivatives were observed in the nuclear fraction and the cytoplasmic fraction is unclear and might be related to how their nuclear localization signals were regulated. In this regard, it is interesting to note that the subcellular distribution of core particles had been shown to be affected by their concentration and cell division (35, 36). Curiously, although in the *trans*-complementation study, the precore protein provided in *trans* could form a chimeric particle with the core protein in the cytoplasm (Fig. 4B and C), it appeared to form primarily precore particles in the nucleus without disrupting the core particle structure (Fig. 4B). In contrast, precore particles were inapparent in the nuclear fraction of cells transfected with the WT-HBV DNA, even though the nuclear fraction also contained a high level of the precore protein derivatives (Fig. 1A and 4B). The reason why the precore protein provided in *trans* preferentially formed precore particles is unclear and might be due to its localization to different compartments in the nucleus, which prohibited it from interaction with the core protein.

A small amount of HBV core particles in the cytoplasm was associated with membranes. This association with membranes was consistent with the previous reports, which indicated that core particles could be assembled on phagophores and were peripherally associated with autophagosomes (17, 18). The core particles in the cytosol of cells transfected by WT-HBV DNA, PCMT DNA, and the core-expressing plasmid all had different mobilities on the gel, further indicating that the precore protein and the HBV DNA genome could affect the structure of core particles (Fig. 3B). Interestingly, based on the relative amounts of the core protein in the cytosol and the membranes, the precore protein and the presence of the HBV DNA genome also appeared to facilitate the association of the core protein with membranes (Fig. 3B). We had previously demonstrated that the precore protein derivatives could be peripherally associated with autophagosomes (18). Thus, it is possible that the precore protein, by forming chimeric particles with the core protein, might enhance the association of the core protein with membranes.

Although multiple core particle structures were identified by the particle gel, in our Southern blot analysis, only the fast-migrating core particles contained the HBV DNA (Fig. 5B). This result was consistent with our observation that only the fast-migrating core particles contained the pgRNA (Fig. 1B and 3C). A previous study found that p22 co-expressed with an HBV genome defective in the production of the core protein could not package the pgRNA (37). This finding was confirmed by a subsequent study, which found that p22 could not fully encapsidate the pgRNA and protect it from RNase digestion (13). Our observation that p22 provided in *trans* could reduce the level of rcDNA (Fig. 5C) argued that, in the presence of the core protein, p22 could be assembled into the core particle to affect HBV DNA replication.

A surprising finding that we made was the increase of the precore protein level by BafA1 but not by MG132. BafA1 suppresses autophagic protein degradation, whereas MG132 is a proteasomal inhibitor. We had previously reported that BafA1 could increase the precore/core protein level in cells transfected with the 1.3mer HBV genomic DNA (18). It was not clear whether this increase was due to the precore protein, the core protein, or both, due to the poor separation of these two proteins on the gel. Our current results indicated that the effect of BafA1 was specific to the precore protein, as BafA1 did not increase the core protein or the HBsAg protein levels. The autophagic degradation of the precore protein did not require its translocation into the ER lumen, as p22 expressed without its signal peptide could also be increased by BafA1. The observation that autophagy might regulate the precore protein levels is interesting and suggests a possible role of autophagy in regulating the biological activities of the precore protein. As the cytosolic precore protein derivatives could suppress HBV DNA replication (Fig. 5C), it is tempting to speculate that HBV used autophagy to reduce the level of cytosolic precore protein derivatives to enhance its replication. Alternatively, p22 had also been shown to bind to toll-like receptor 2 (TLR2) to reduce its level (38). It is thus also possible that HBV might use p22 and autophagic degradation to remove TLR2.

## MATERIALS AND METHODS

### Cell cultures and DNA constructs

Huh7 cells were cultured at 37°C and 5% CO_2_ in Dulbecco's Modified Eagle Medium (DMEM) (Corning) supplemented with 10% fetal bovine serum (FBS) (Gibco), and HepAD38 cells were cultured in DMEM + F12 with 10% FBS. The plasmid WT-HBV, also known as p1.3×HBV, carried the 1.3mer overlength HBV genomic DNA of genotype A (adw2 subtype) in the pUC19 vector (39). pUC19 was thus used as a control plasmid in this study. The plasmid PCMT, also known as p1.3×HBV PC^–^, contained a 1.3mer HBV genome with a G to A mutation at nucleotide 1896, which converted codon 28 of the precore sequence from TGG to TAG and abolished the expression of the precore protein. A compensatory mutation of C to U at nucleotide 1858 was also generated to maintain the stem-loop structure of the epsilon packaging signal (23). Other HBV protein expression plasmids used in this study, including pCMV-precore, pCMV-core, and pcDNA-p22, had been described before (18, 40). The plasmid pCMV-precore contained the HBV sequence from nucleotide 1757 to the XbaI site of the adw2 genome and included the entire precore protein-coding sequence, whereas the plasmid pCMV-core was almost identical to pCMV-precore with the exception that the HBV sequence in pCMV-core started at nucleotide 1821, one nucleotide downstream of the precore start codon (40). The plasmid pcDNA-p22 was constructed by deleting the signal peptide-coding sequence (amino acids 2–19) from pCMV-precore (18). The expressions of precore, core, and p22 were all driven by the immediate early promoter of the CMV.

### DNA transfection and drug treatments

The DNA transfection (15 µg per 10 cm dish) was conducted using Lipofectamine 3000 (Invitrogen) following the manufacturer’s protocol. For the *trans*-complementation studies, the amounts of DNA plasmids used for the transfection of cells in two 10 cm dishes are shown in Fig. 4A and 5C. For drug treatments, cells in a 10 cm dish were transfected with 10 µg DNA and then treated with 80 nM of bafilomycin A1 (Sigma SML1661) or the vehicle dimethyl sulfoxide (DMSO) for 16 hours at 32 hours after the DNA transfection or with 2 µM of MG132 (Sigma M7449) or DMSO for 4 hours at 44 hours after the DNA transfection. Cells were then harvested 48 hours after transfection for immunoblot analysis.

### Antibodies

The commercial primary antibodies used in this study included the mouse anti-HBcAg antibody (Abcam ab8637), rabbit anti-alpha tubulin antibody (Proteintech 66031-1-Ig), rabbit anti-lamin B1 antibody (Abcam ab16048), mouse anti-GAPDH antibody (Proteintech 60004-1-Ig), rhodamine-conjugated anti-GAPDH antibody (Bio-Rad 12004168), rabbit anti-ATG5 antibody (Cell Signaling 2630S), rabbit anti-LC3B antibody (Sigma L7543), rabbit anti-CD63 antibody (Proteintech 25682-1-AP), rabbit anti-GM130 antibody (Proteintech 11308-1-AP), and rabbit anti-HBsAg antibody (Novus Biologicals NB100-62652). Rabbit antibodies targeting HBeAg were prepared in our own laboratory and had been described previously (20). In short, the anti-HBeAg was generated by immunizing rabbits with core particles expressed in *Escherichia coli*. Prior to injection, the core particles were denatured with sodium dodecyl sulfate (SDS). This anti-HBeAg was used for immunoblot analysis of precore/core proteins separated by SDS-PAGE. The Abcam anti-HBcAg was used for non-denaturing agarose gel electrophoresis (NAGE) analysis of core particles. Secondary antibodies used in our studies were anti-rabbit and anti-mouse antibodies conjugated with horseradish peroxidase (HRP) (Abcam 6721 and 6728).

### ELISA for HBeAg

The incubation media from WT-HBV DNA-transfected Huh7 cells or HepAD38 cells 1 day after passage were harvested for HBeAg analysis using an ELISA kit following the manufacturer’s protocol (International ImmunoDiagnostics). The medium was diluted 10-fold with phosphate-buffered saline (PBS), and 100 µL of the diluted samples was used for the assay. All assays were conducted in triplicate. A serial dilution of the calibrator was also analyzed to generate a standard curve, which was used to convert the absorbance at OD450 to Paul-Ehrlich-Institut (PEI) units.

### Immunoblot analysis

Depending on the experiments, Huh7 cells were routinely lysed 48 hours after DNA transfection using either Radioimmunoprecipitation Assay (RIPA) Lysis and Extraction Buffer (ThermoFisher) or a hypotonic buffer (10 mM Tris-HCl [pH 7.5], 10 mM KCl, 5 mM MgCl_2_) with the protease inhibitor cocktail (Roche) for SDS-polyacrylamide gel electrophoresis (SDS-PAGE) or NAGE. For HepAD38 cells, confluent cells were lysed 1 day after passage for analysis. After removing cell debris by a brief centrifugation, the lysates were boiled in 1× Laemmli buffer (Bio-Rad) and subjected to SDS-PAGE using 10%, 12%, or 15% gel (Bio-Rad). Alternatively, cell lysates were treated with 0.1% Nonidet P-40 Substitute (G-Biosciences) before loading onto 1% agarose gel (Promega) for electrophoresis. The proteins were then transferred to a polyvinylidene difluoride membrane (Bio-Rad) or the nitrocellulose (NC) membrane (Cytiva). The membrane was blocked with 5% blotting-grade blocker (Bio-Rad) made by dissolving nonfat milk in Tris-buffered saline (TBS) (10 mM Tris-HCl [pH 7.5], 150 mM NaCl) containing 1% Tween 20 (TBST) for 30 minutes. Afterward, the membrane was washed three times using TBST and incubated with the primary antibody in 5% blotting-grade blocker for 3hours at room temperature or overnight at 4°C. Following three washes with TBST, the membrane was incubated with HRP-conjugated secondary antibody for 1 hour or with rhodamine-conjugated anti-GAPDH antibody for 2 hours at room temperature. After three washes with TBST, SuperSignal West Pico PLUS Chemiluminescent Substrate (ThermoFisher) containing 1% SuperSignal West Femto Maximum Sensitivity Substrate (ThermoFisher) was added to the membrane and incubated for 1 minute. The image was captured using the ChemiDoc MP Imaging System (Bio-Rad).

### Subcellular fractionation

The cytoplasmic fraction and the nuclear fraction were isolated using the NuCLEAR Extraction Kit (Sigma) following the manufacturer’s instructions. Briefly, cells grown in three 10 cm plates were washed and then lysed with 500 µL of hypotonic buffer containing the protease inhibitor cocktail and passed through a 27-gauge needle of a 1 mL syringe (BD) at least 20 times. After centrifugation at 1,000 × *g* for 5 minutes at 4°C, the supernatant (i.e., the cytoplasmic lysates) was transferred to a new tube, and NP-40 was added to a final concentration of 0.6%. The crude nuclear pellet was washed three times with the hypotonic buffer and then suspended in 75 µL of hypotonic buffer (the same volume as the pellet) containing the protease inhibitor cocktail and 0.6% NP-40. To disrupt the nuclei, the pellet was initially pipetted 20 times and incubated in the ice for 1 hour. During the incubation, the pellet was pipetted 20 times every 5 to 10 minutes and continuously pipetted for 2 minutes at the end of the incubation. The residual cell debris was subsequently removed by centrifugation at 16,000 × *g* for 30 minutes at 4°C, and the supernatant (i.e., the nuclear extracts) was harvested. For immunoblot analysis, 5 µL of the 500 µL cytoplasmic lysates or 25 µL of the 75 µL nuclear extracts was used.

The separation of membranes and the cytosol was conducted using the membrane flotation assay as previously described (18, 41). In brief, the cytoplasmic lysates were isolated as described above using the hypotonic buffer without the addition of NP-40. The supernatant was mixed with 4.5 mL 80% sucrose solution in the low-salt buffer (LSB) (50 mM Tris-HCl [pH 7.5], 25 mM KCl, and 5 mM MgCl_2_) and overlaid first with 4 mL 55% sucrose solution in LSB and next with 3.5 mL 10% sucrose solution in LSB. The discontinuous sucrose gradient was centrifuged at 38,000 rpm in a Beckman SW40Ti rotor for 18 hours at 4°C, without using the brake during the deceleration. Following centrifugation, 1 mL fractions were collected from the top of the gradient, and 20 µL of each fraction was used for immunoblot analysis.

### NAGE

The HBV core particles were analyzed by NAGE as previously described (42). Briefly, 20 µL of different samples in 1× DNA loading buffer (10 mM EDTA [pH 8.0], 50% (vol/vol) glycerol, 0.25% (wt/vol) bromophenol blue, and 0.25% (wt/vol) xylene cyanol) was loaded into the well of the 1% agarose gel for electrophoresis. All the samples, including the cytoplasmic fraction, nuclear fraction, and sucrose gradient fractions, were treated with 0.6% NP-40 to remove membranes prior to NAGE. Meanwhile, the NC membrane was incubated first in distilled water for 5 minutes and then in the TNE buffer (100 mM Tris-HCl [pH 7.4], 1.5 M NaCl, and 10 mM EDTA) for 15 minutes. The particles were then transferred from the gel to the NC membrane using the upward capillary transfer method in the TNE buffer overnight. The membrane was subjected to the immunoblot analysis or Southern blot as previously described (42). For RNase treatment, 20 µL of the samples was treated with 200 µg/mL RNase A (New England Biolabs) at 37°C for 30 minutes before NAGE. For Northern blot analysis, the cytoplasmic fraction and the nuclear fraction were each split equally into two aliquots, one with and the other without the RNase treatment. The polyethylene glycol (PEG) was then added to the samples to a final concentration of 10%, and the tubes containing the samples were rocked for at least 6 hours at 4°C using a HulaMixer Sample Mixer (Invitrogen). After the centrifugation at 10,000 × *g* for 30 minutes at 4°C, the pellet was resuspended in 20 µL TNE buffer containing 1× DNA loading buffer and incubated overnight at 4°C. The samples were then subjected to NAGE as described above. After the transfer, the membrane was baked at 80°C for 90 minutes. For Southern blot analysis, PEG was added to 300 µL of the membrane fraction or the cytosolic fraction to a final concentration of 10%. The same procedures were then conducted as described above for the Northern blot analysis, with the exception that after the transfer, the membrane was incubated in the denaturing solution (0.5 M NaOH, 1.5 M NaCl) for 15 minutes and then in the neutralization solution (1.5 M NaCl, 1 M Tris-HCl [pH 7.4]) for 5 minutes before baking.

The antisense riboprobe for Northern blot analysis was prepared by run-off transcription using 1 µg of the HindIII-digested pCMV-precore plasmid as the template. The transcription was conducted using MEGAscript SP6 Transcription Kit (Invitrogen) and Digoxigenin (DIG) RNA Labeling Mix (Roche), which contained DIG-11-UTP. The probe was then purified by MEGAclear Transcription Clean-Up Kit (Invitrogen). The DNA probe for Southern blot was prepared by random priming using 1 µg of PvuII-digested WT-HBV plasmid as the template and labeled with DIG-11-dUTP for at least 20 hours at 37°C according to the manual of the DIG-High Prime DNA Labeling and Detection Starter Kit I (Roche). The hybridization and immunological detection were conducted as described in the manual. For hybridization, 25 ng/mL DIG-labeled riboprobe or DNA probe was added to DIG Easy Hyb buffer and incubated with the membrane overnight at 48°C. The following day, the membrane was washed sequentially in 2× saline-sodium citrate (SSC) (20× SSC: 3 M NaCl, 0.3 M sodium citrate) containing 0.5% SDS for 15 minutes twice, 0.5× SSC + 0.5% SDS for 15 minutes once, and 0.15× SSC + 0.5% SDS for 15 minutes once at 68°C. The immunological detection was conducted following the manual of the DIG Wash and Block Buffer Set (Roche) with 75 mU/mL of alkaline phosphatase-conjugated anti-DIG antibody. Finally, the color substrate nitro-blue tetrazolium chloride/5-bromo-4-chloro-3′-indolyphosphate p-toluidine salt was incubated with the membrane from 1 hour to overnight at room temperature for the development of the signals. The image was captured using the ChemiDoc MP Imaging System.

### Southern blot analysis of intracellular HBV DNA in core particles

To extract intracellular HBV DNA that was packaged in the core particles, sterile water was added to cytoplasmic lysates, the nuclear extracts, or sucrose gradient fractions to a final volume of 500 µL. The free DNA (i.e., plasmid DNA) in the samples was then digested with 20 U/mL of Turbo DNase (ThermoFisher) and 2,000 gel units/mL of micrococcal nuclease (New England Biolabs) for 1 hour at 37°C. The reaction was stopped by the addition of EDTA to a final concentration of 50 mM. The samples were then incubated with 30 ng/µL of proteinase K for at least 6 hours at 56°C. Subsequently, the HBV DNA was isolated by phenol-chloroform extraction and ethanol precipitation. Due to the interference of sucrose on phase separations during the phenol-chloroform extraction, the sucrose gradient samples were first precipitated with 10% PEG overnight at 4°C. The next day, after centrifugation at 16,000 × *g* for 30 minutes at 4°C, the pellet was resuspended in 100 µL HBV DNA lysis buffer (20 mM Tris-HC [pH 7.5], 20 mM EDTA, 50 mM NaCl, 0.5% SDS, and 30 ng/µL of proteinase K) and incubated for at least 6 hours at 56°C before phenol-chloroform extraction. The DNA pellet was rinsed with 70% ethanol and resuspended in Tris-EDTA (TE) buffer for Southern blot analysis as described above.

### Statistical analysis

All statistical analyses were performed using Microsoft Excel software. The statistical significance was analyzed using the Student’s *t*-test, in which *** indicated *P* < 0.001, ** indicated *P* < 0.01, and * indicated *P* < 0.05. NS indicated *P* > 0.05.

## Data Availability

The original files of the blots, which include immunoblots, Northern blots, and Southern blots, are provided in the supplemental material.

## SUPPLEMENTAL MATERIAL

Figure S1 mbio.03972-24-s0001.pdf Electron micrograph of recombinant yeast core particles.

Figure S2 mbio.03972-24-s0002.pdf Analysis of core particles in HepAD38 cells.

Figure S3 mbio.03972-24-s0003.pdf A longer exposure of Fig. 4B.

Legends mbio.03972-24-s0004.docx Supplemental figure legends.

Original Blots mbio.03972-24-s0005.pdf Original images of blots before assembly into figures.

ASM does not own the copyrights to Supplemental Material that may be linked to, or accessed through, an article. The authors have granted ASM a non-exclusive, world-wide license to publish the Supplemental Material files. Please contact the corresponding author directly for reuse.
