# Supplementary figures and images for: Effects of cellular membranes and the precore protein on hepatitis B virus core particle assembly and DNA replication
Source: mBio. 2025 Mar 5;16(4):e03972-24. doi: 10.1128/mbio.03972-24 (PMC11980540; doi:10.1128/mbio.03972-24)

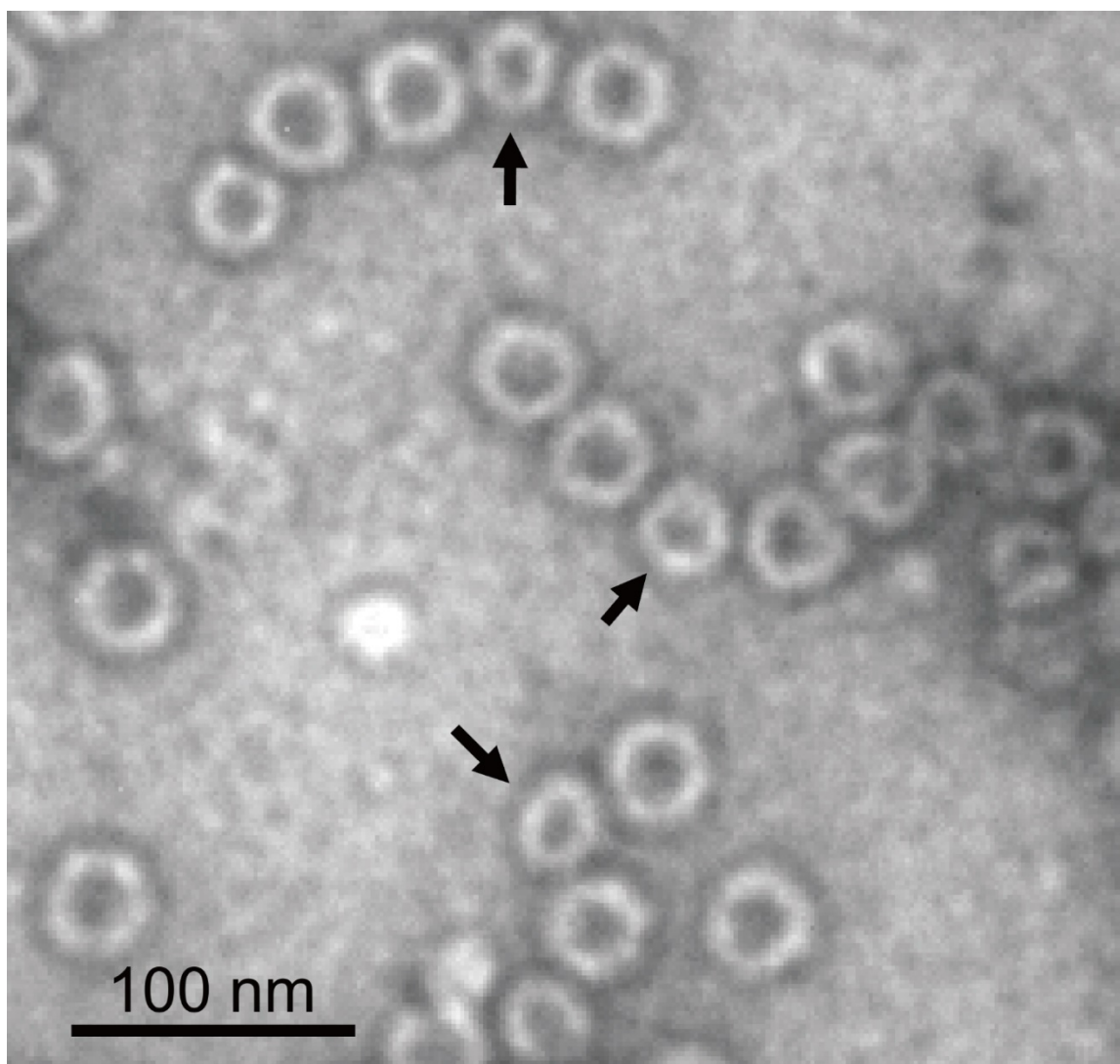

Supplement: Figure S1 — Electron micrograph of recombinant yeast core particles. [file mbio.03972-24-s0001.pdf]

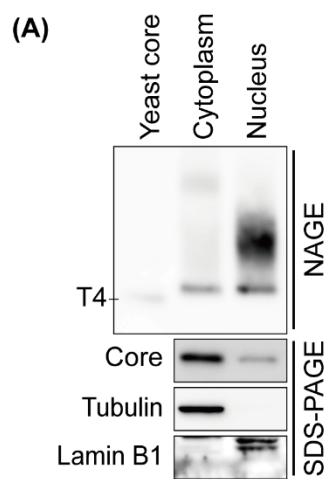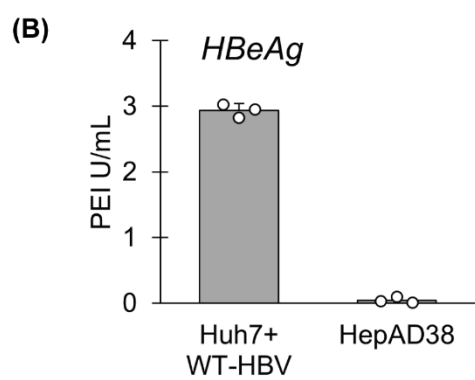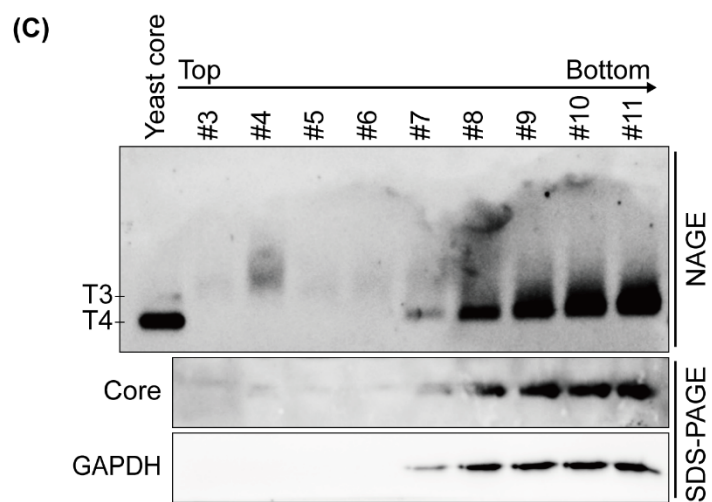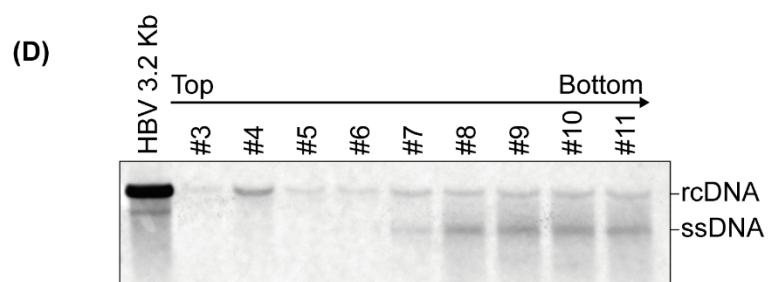

Supplement: Figure S2 — Analysis of core particles in HepAD38 cells. [file mbio.03972-24-s0002.pdf]

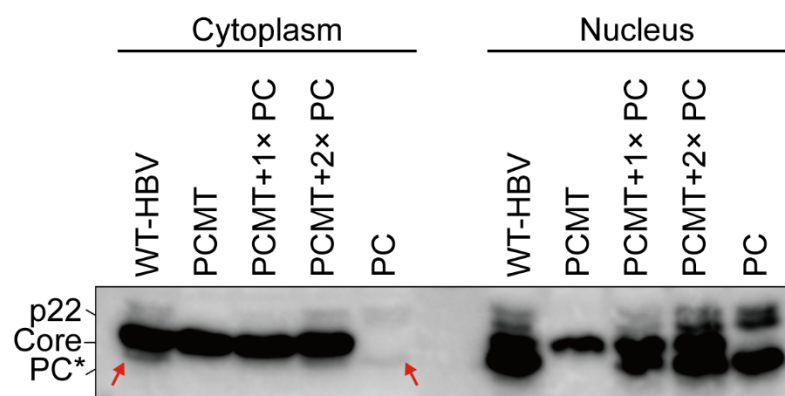

Supplement: Figure S3 — A longer exposure of Fig. 4B. [file mbio.03972-24-s0003.pdf]
