# Supplementary material for: Effects of cellular membranes and the precore protein on hepatitis B virus core particle assembly and DNA replication
Source: mBio. 2025 Mar 5;16(4):e03972-24. doi: 10.1128/mbio.03972-24 (PMC11980540; doi:10.1128/mbio.03972-24)
Supplement: Legends — Supplemental figure legends. [file mbio.03972-24-s0004.docx]

**SUPPLEMENTAL FIGURE LEGENDS**

**Figure S1. Electron micrograph of recombinant yeast core particles.** The yeast core particles were a generous gift of Dr. David Chien (ImmuOn Therapeutics). They were produced using the full-length HBV core protein (genotype A). The particles were negatively stained with 5% uranyl acetate for electron microscopy. Arrows denote the minor population of core particles that are smaller in size and may represent the T = 3 structure.

**Figure S2. Analysis of core particles in HepAD38 cells.** (A) The cytoplasmic fraction and the nuclear fraction of HepAD38 cells were isolated as described in the Fig. 1 legend for core particle and immunoblot analyses. (B) ELISA analysis for HBeAg in the incubation media of Huh7 cells transfected with the WT-HBV DNA or HepAD38 cells. The study was conducted in triplicate. (C) The cytoplasmic fraction of HepAD38 cells was further fractionated in a discontinuous sucrose gradient followed by particle gel analysis and immunoblot analysis. (D) Southern-blot analysis of core particle-associated HBV DNA in different fractions of the sucrose gradient. A 3.2kb HBV genomic DNA was used as the marker. In (C) and (D), fraction 4 is the membrane fraction.

**Figure S3. A longer exposure of Fig. 4B.** Red arrows denote the precore protein derivatives.
