# Supplementary material for: Effects of cellular membranes and the precore protein on hepatitis B virus core particle assembly and DNA replication
Source: mBio. 2025 Mar 5;16(4):e03972-24. doi: 10.1128/mbio.03972-24 (PMC11980540; doi:10.1128/mbio.03972-24)

**Legend for Original Blots and Images**

This file contains the original images of the blots before they were assembled into various figures that include Figure 1A, 1B, 1C, 2A, 2B, 3A, 3B, 3C, 4B, 4C, 5A, 5B, 5C, 5D, 6A, 6C and the three supplemental figures.

Fig. 1A

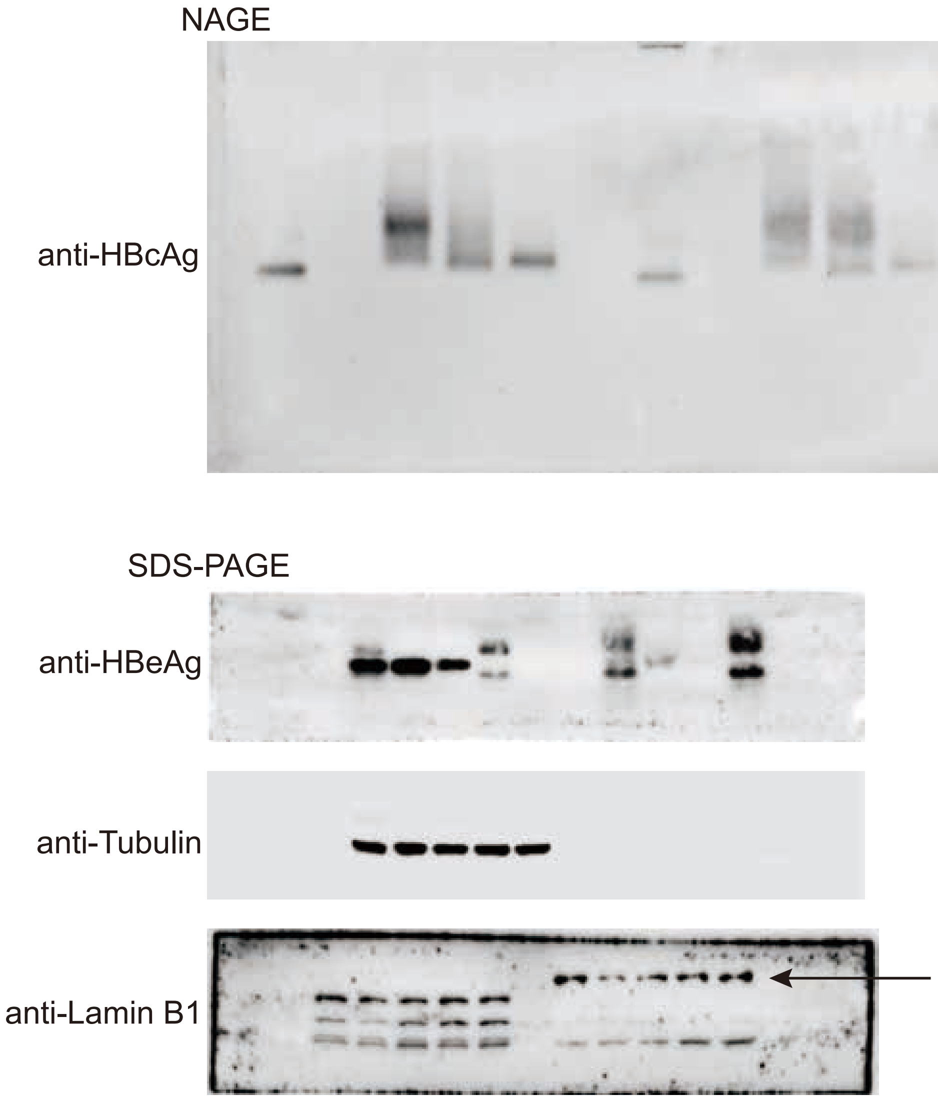
NAGE
anti-HBcAg
SDS-PAGE
anti-HBeAg
anti-Tubulin
anti-Lamin B1

Fig. 1B

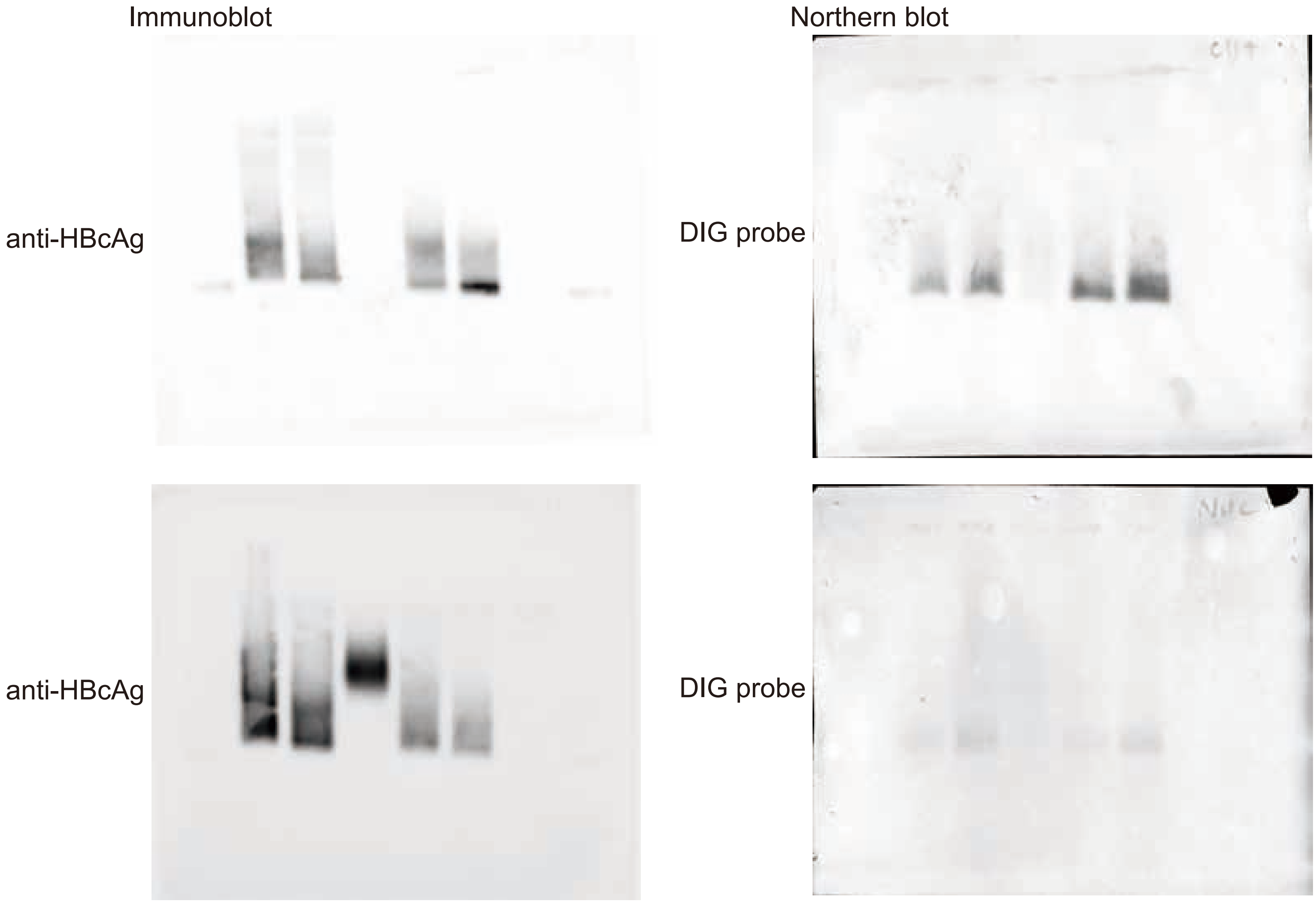
Immunoblot
Northern blot
anti-HBcAg
DIG probe
anti-HBcAg
DIG probe

Fig. 1C

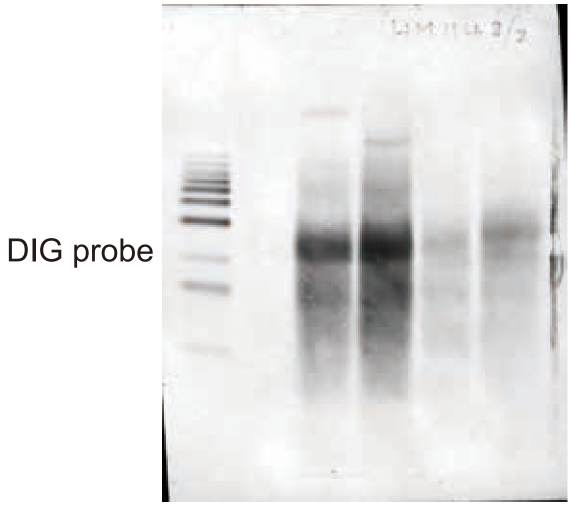
DIG probe

Fig. 2A

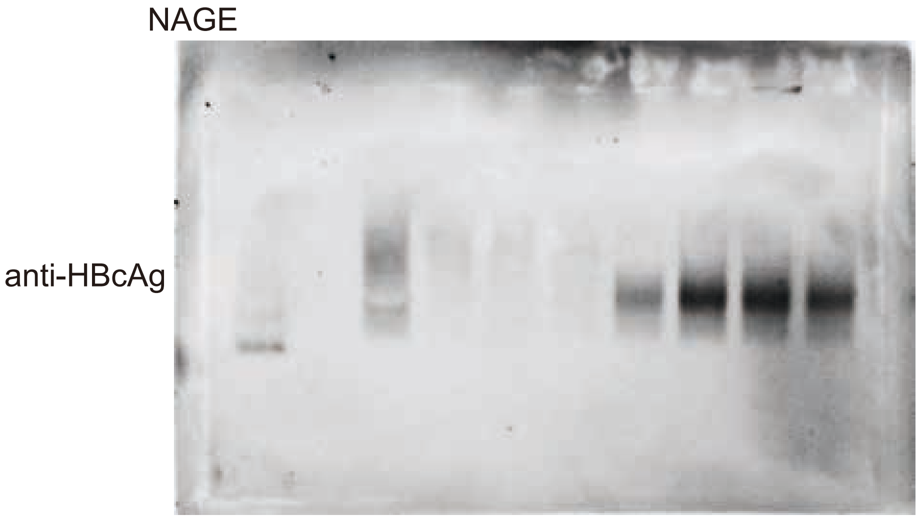
NAGE
anti-HBcAg

Fig. 2B

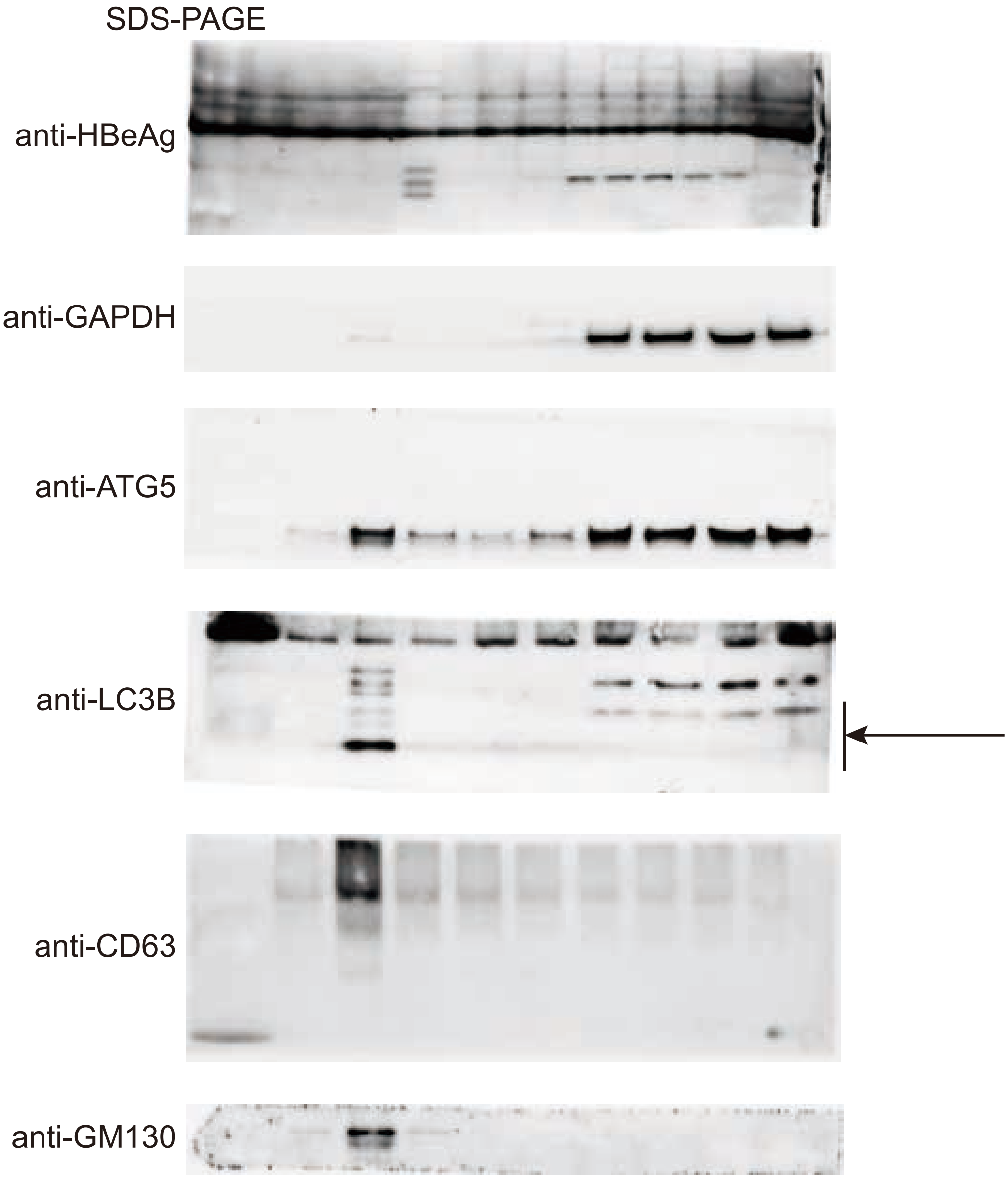
SDS-PAGE
anti-HBeAg
anti-GAPDH
anti-ATG5
anti-LC3B
anti-CD63
anti-GM130

Fig. 3A

pUC19

anti-HBcAg

PCMT

anti-HBcAg

anti-HBeAg

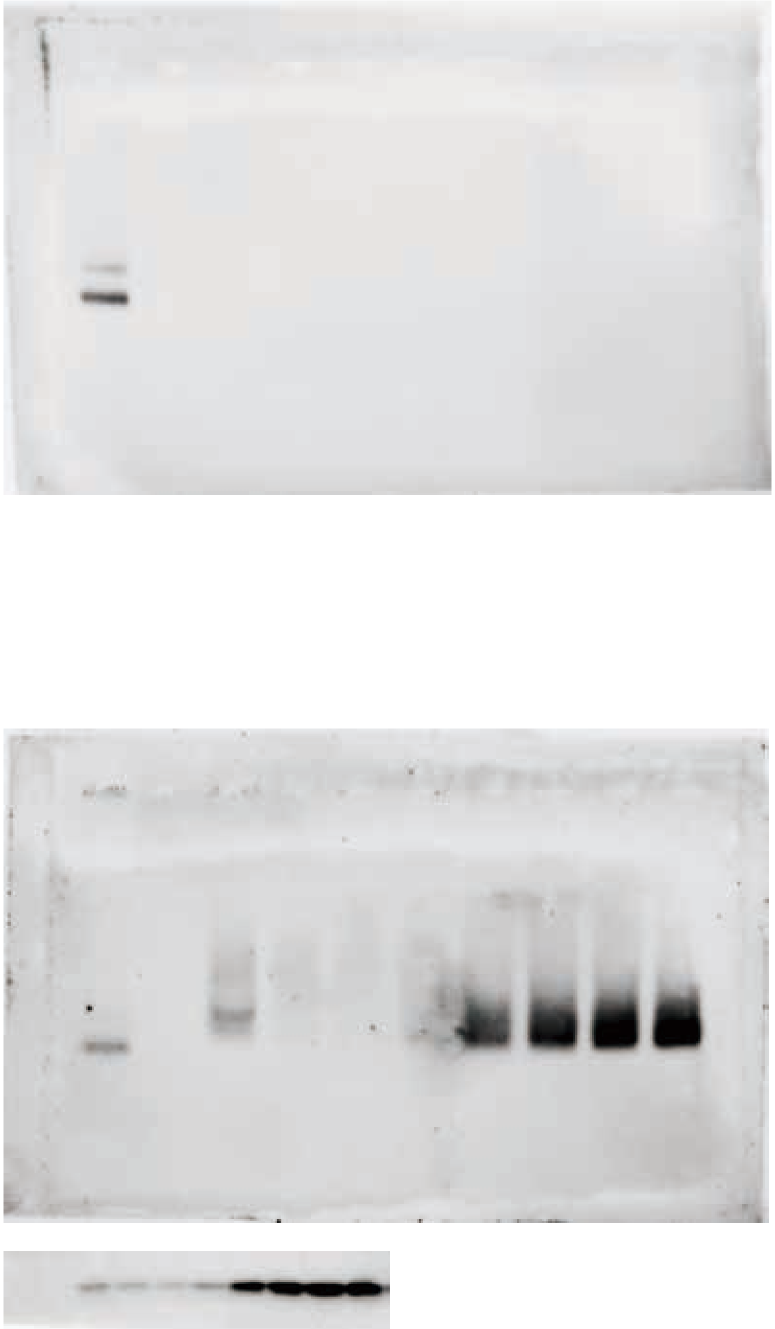

Fig. 3A

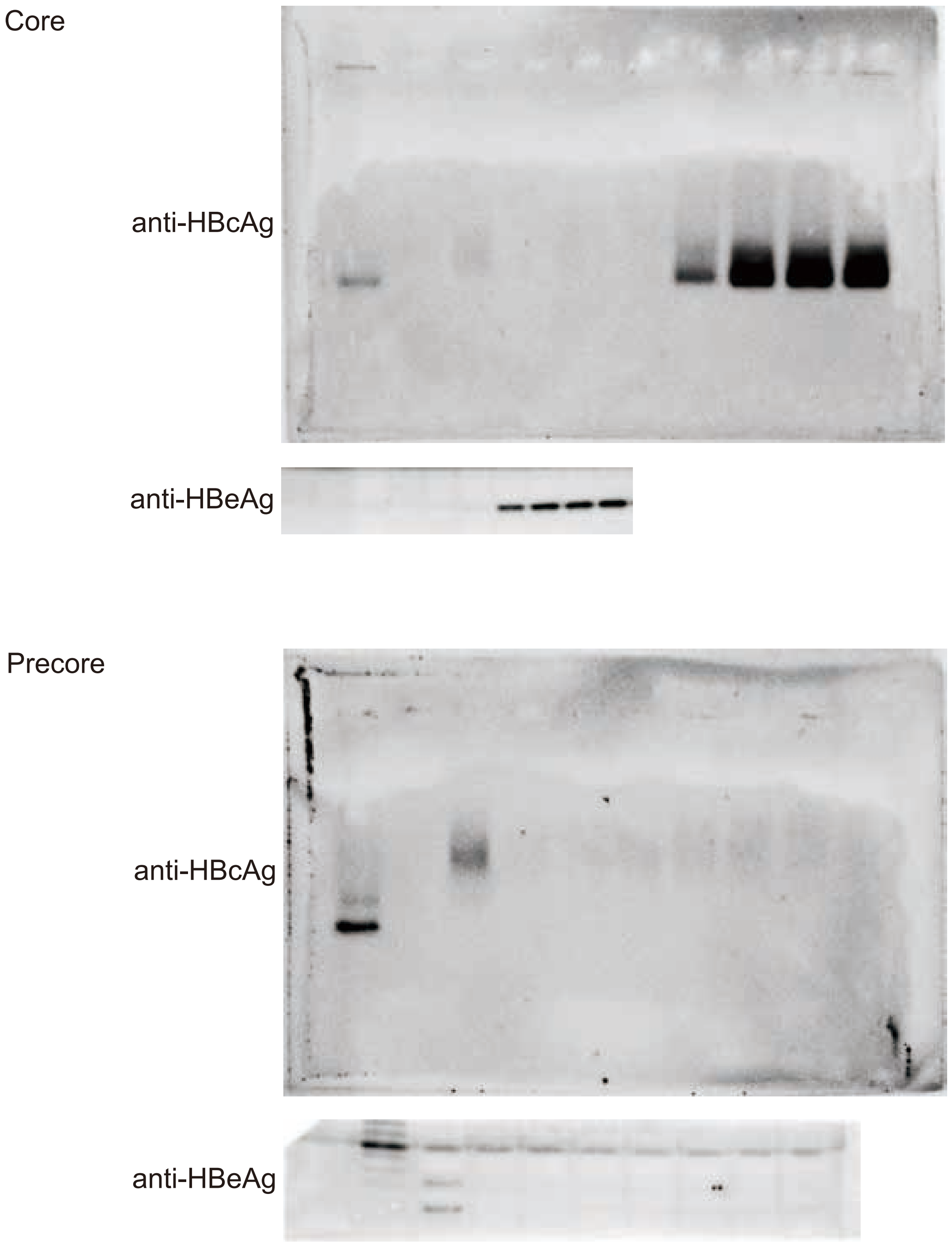

Fig. 3B

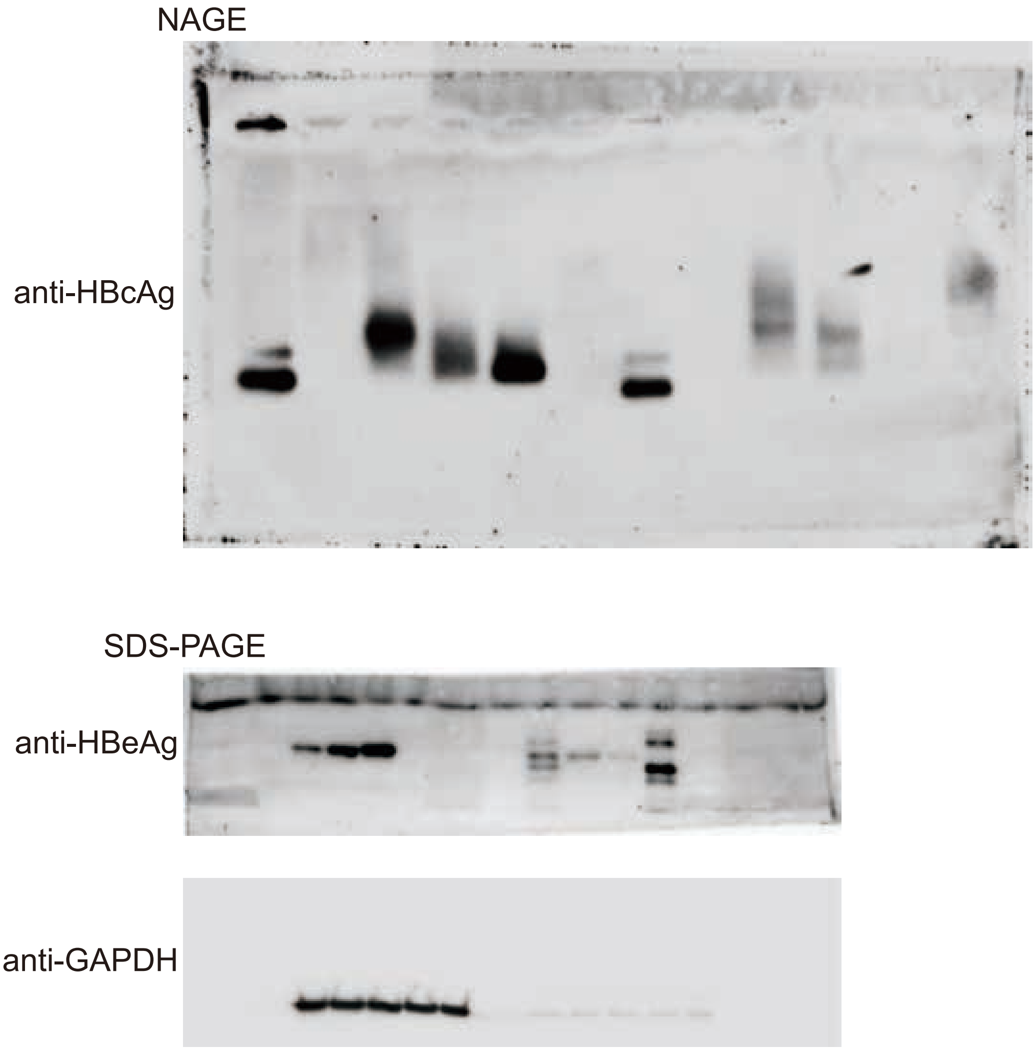

Fig. 3C

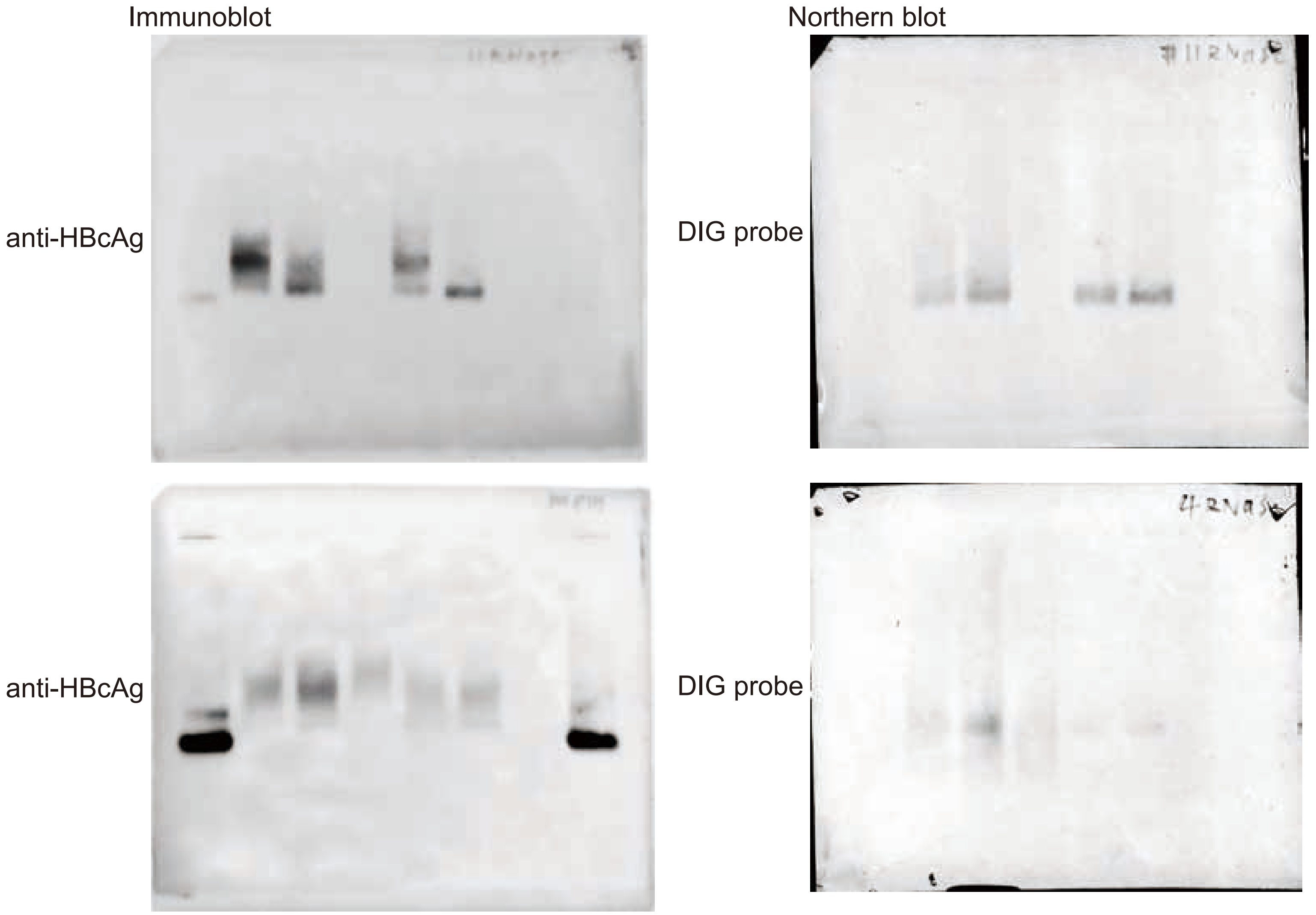
Immunoblot
Northern blot
anti-HBcAg
DIG probe
anti-HBcAg
DIG probe

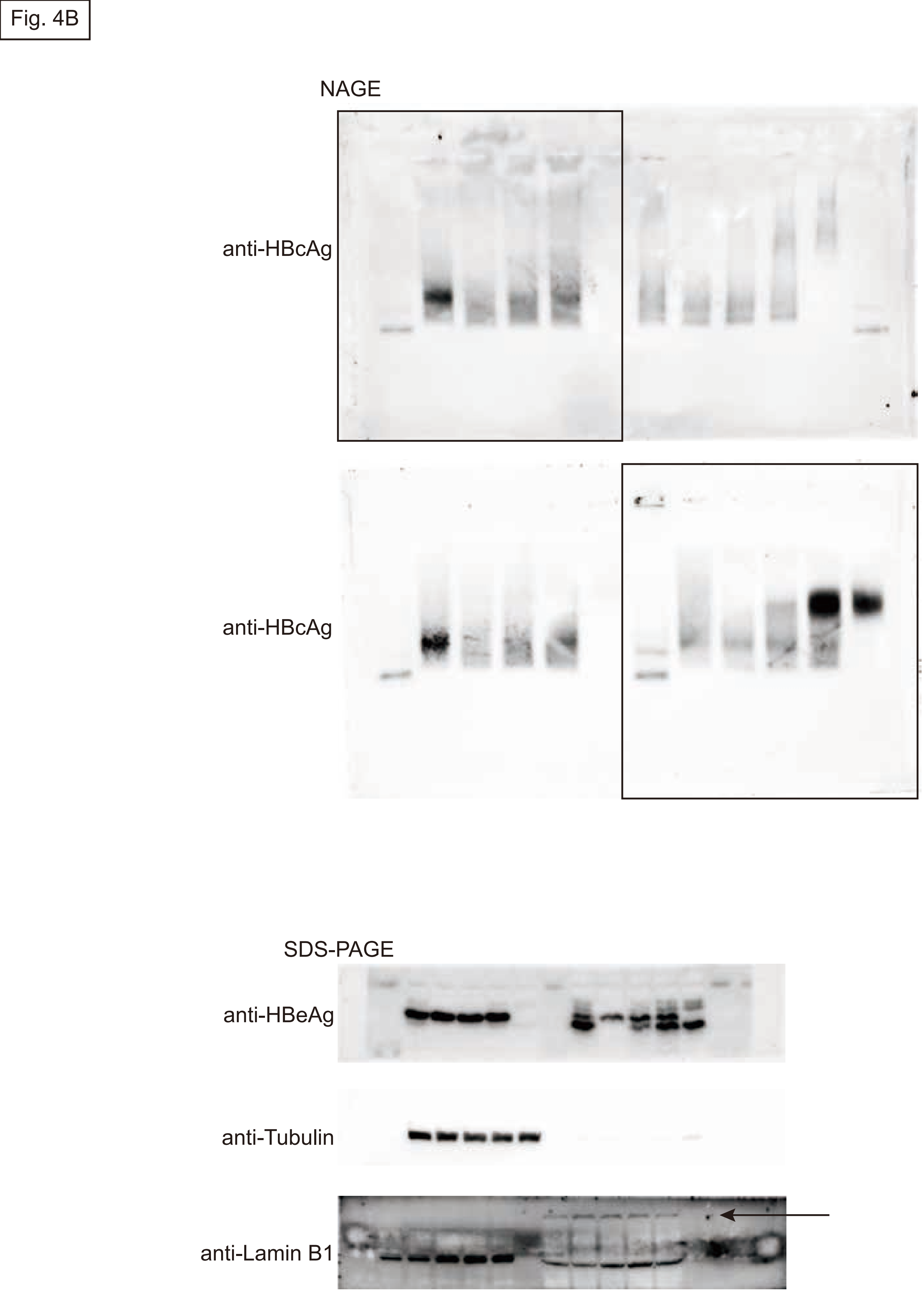
Fig. 4B
NAGE
anti-HBcAg
anti-HBcAg
SDS-PAGE
anti-HBeAg
anti-Tubulin
anti-Lamin B1

Fig. 4C

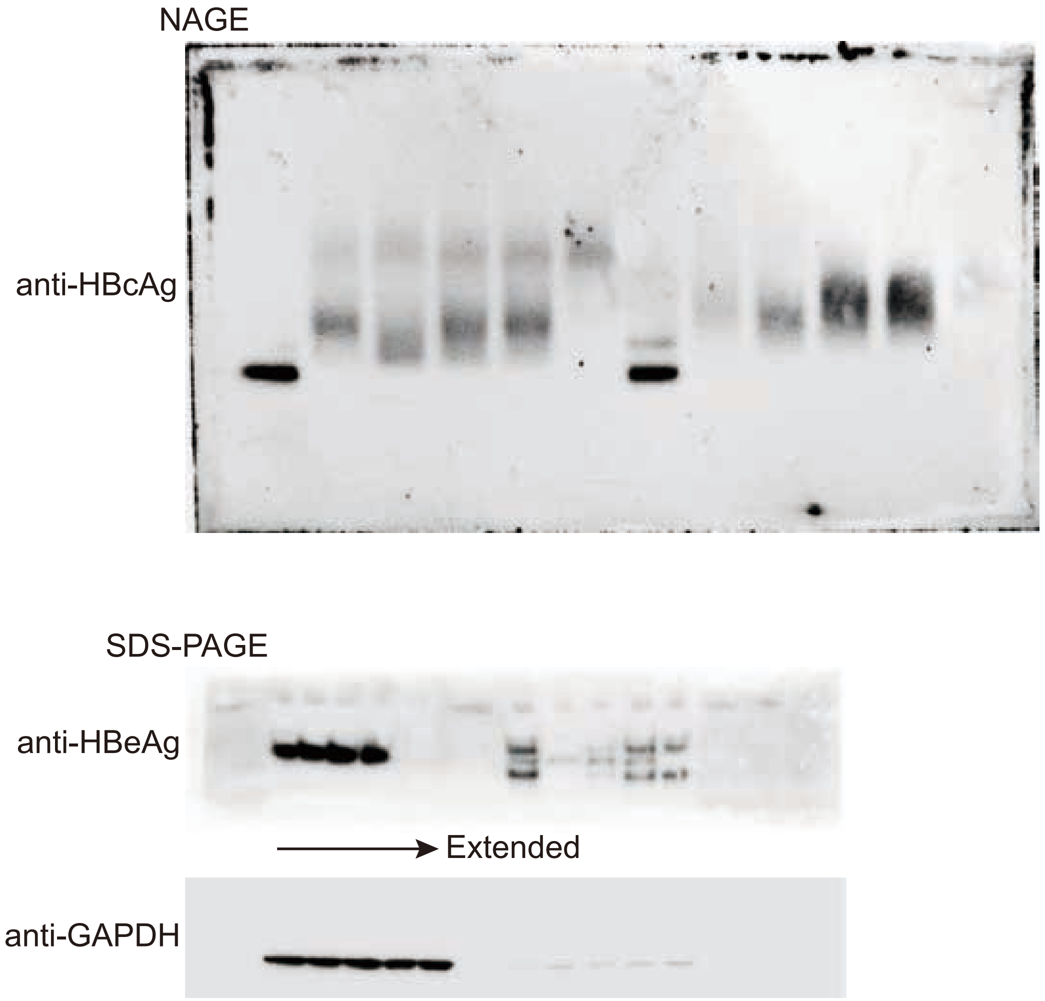

Fig. 5A

Southern blot

WT-HBV

PCMT

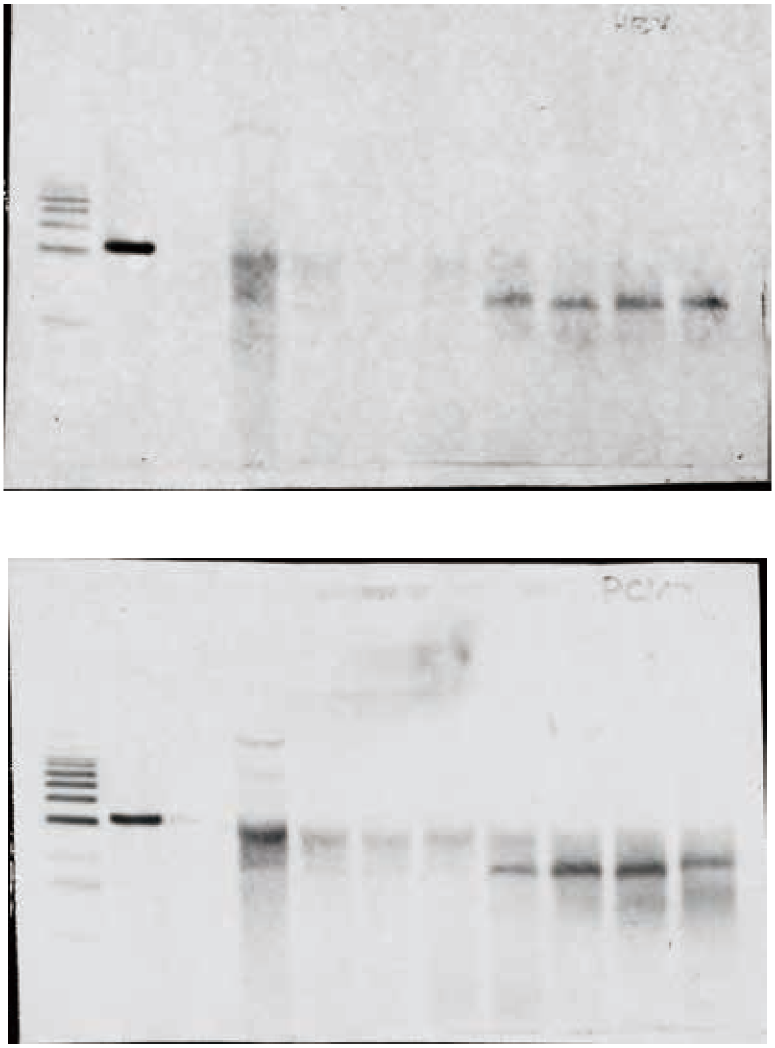

Fig. 5B

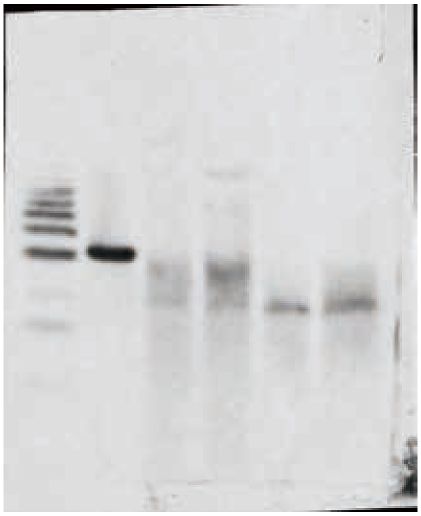

Fig. 5C

Southern blot

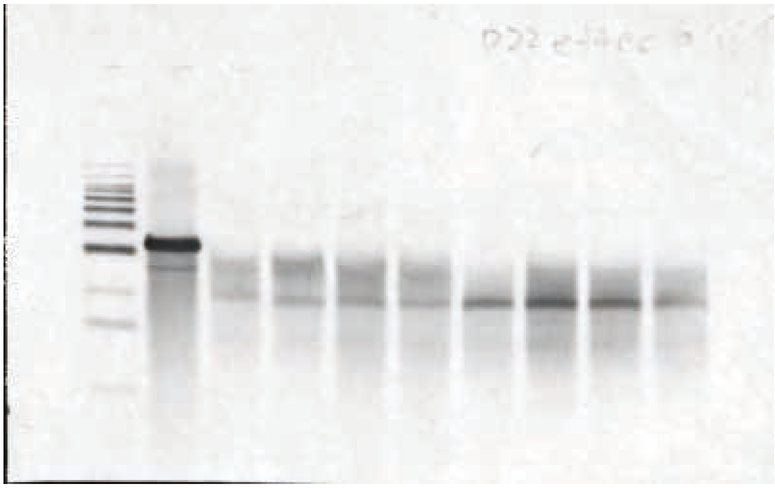

Fig. 5D

Immunoblot

anti-HBcAg

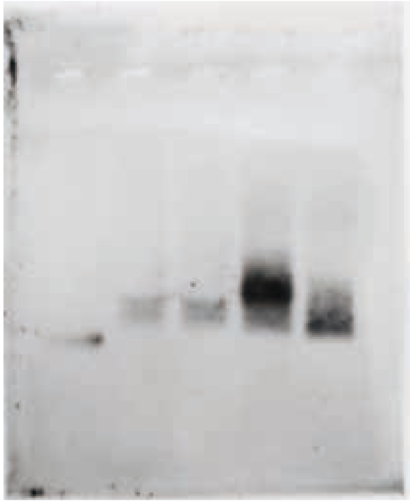

Southern blot

DIG probe

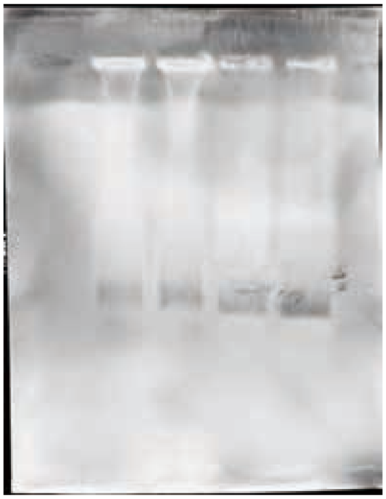

Fig. 6A

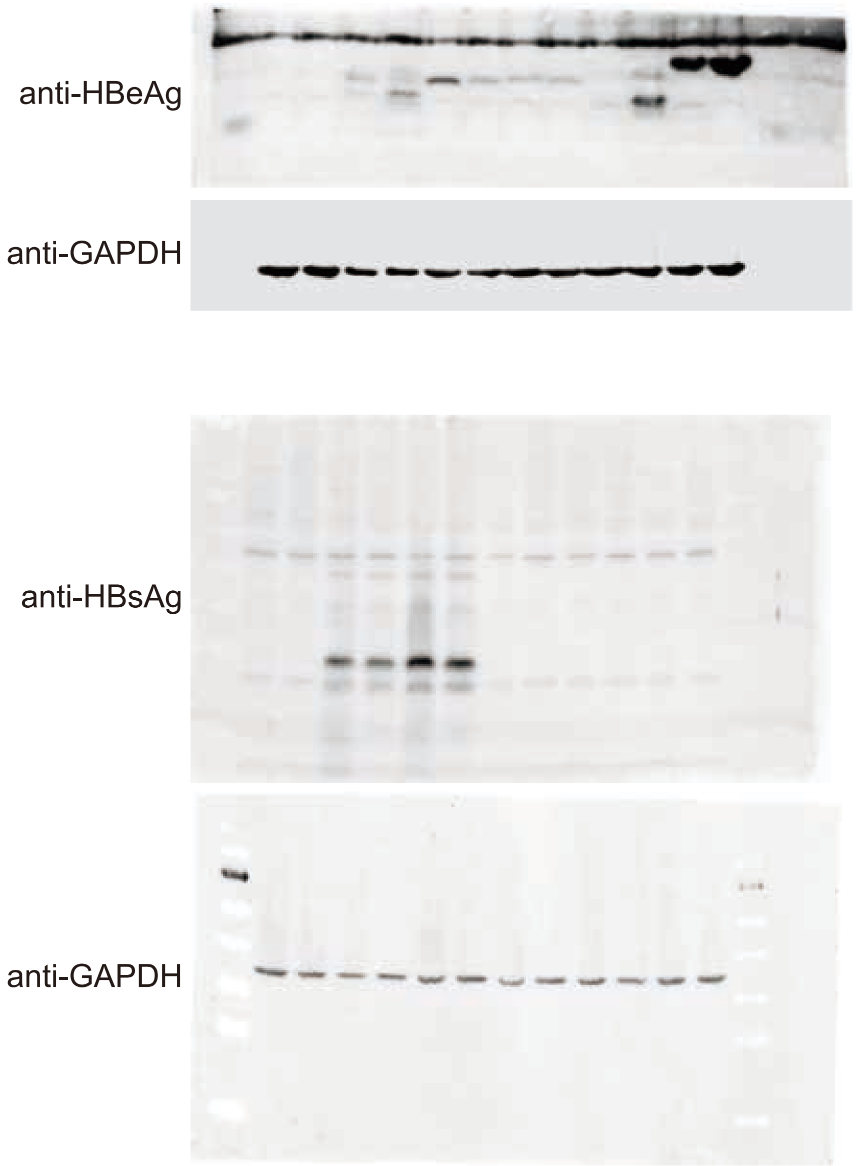

Fig. 6C

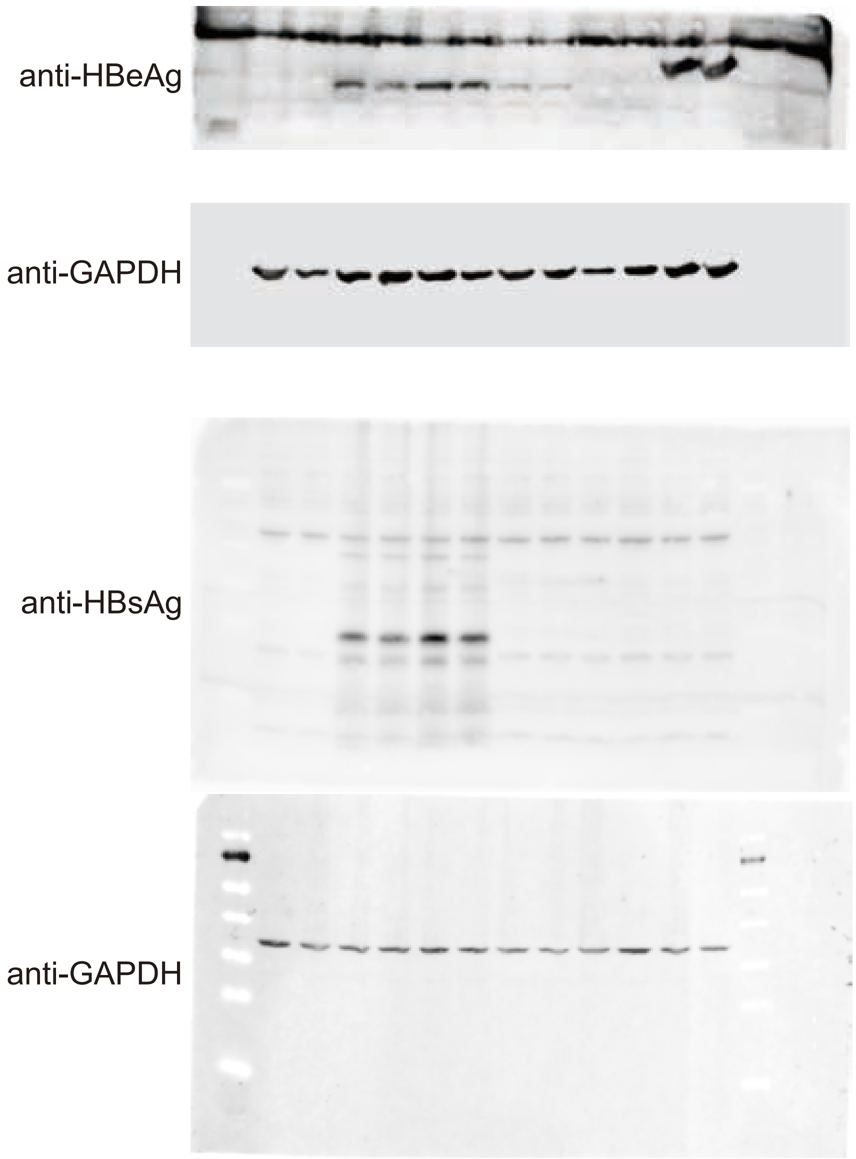

Fig. S1

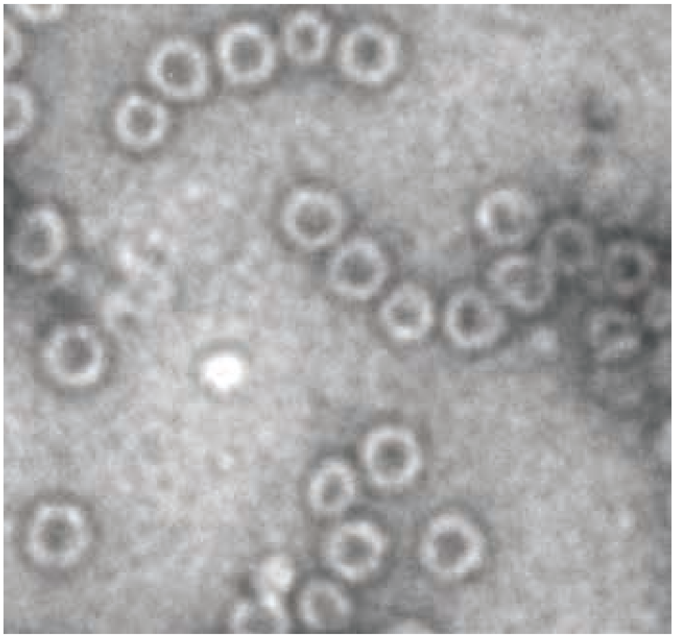

Fig. S2A

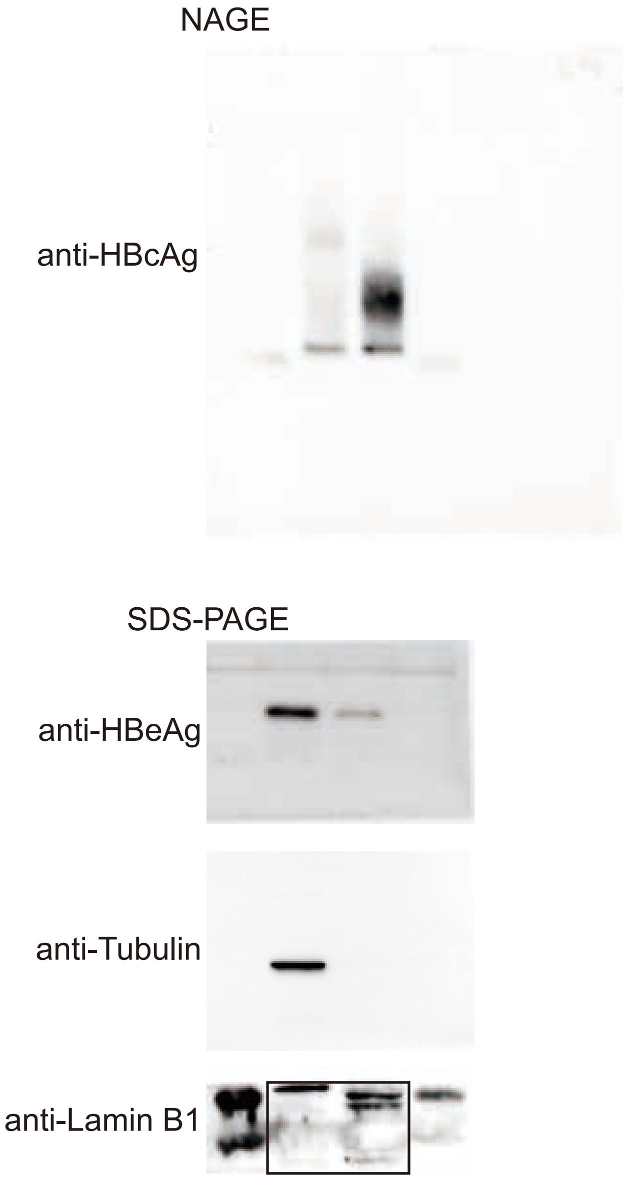
NAGE
anti-HBcAg
SDS-PAGE
anti-HBeAg
anti-Tubulin
anti-Lamin B1

Fig. S2C

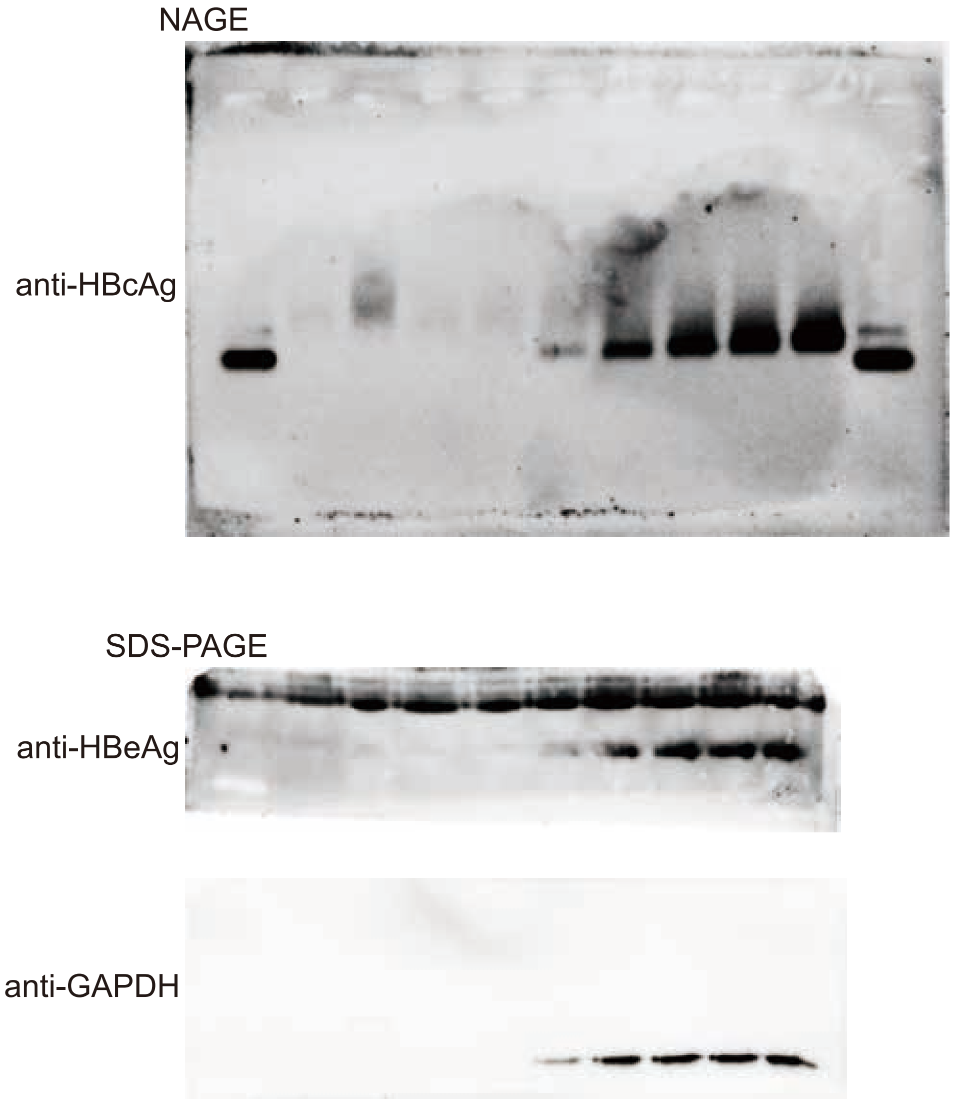


Fig. S2D

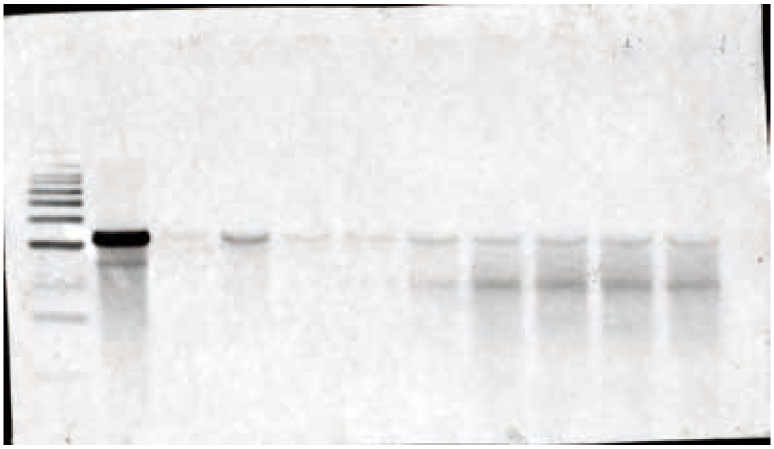

Fig. S3

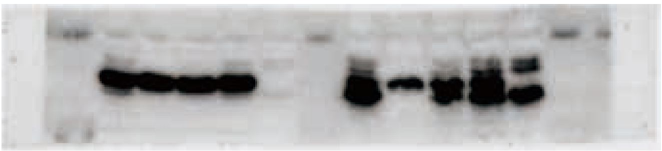

Supplement: Original Blots — Original images of blots before assembly into figures. [file mbio.03972-24-s0005.pdf]
